# Combatting insects mediated biotic stress through plant associated endophytic entomopathogenic fungi in horticultural crops

**DOI:** 10.3389/fpls.2022.1098673

**Published:** 2023-01-19

**Authors:** Ipsita Samal, Tanmaya Kumar Bhoi, Prasanta Kumar Majhi, Sneha Murmu, Asit Kumar Pradhan, Dilip Kumar, Varun Saini, Amit Umesh Paschapur, M Nikhil Raj, Ankur, Suryakant Manik, Partha Pratim Behera, Deepak Kumar Mahanta, J. Komal, Pravej Alam, Thamer Al Balawi

**Affiliations:** ^1^ Department of Entomology, Sri Sri University, Cuttack, Odisha, India; ^2^ Forest Protection Division, Indian Council of Forestry Research and Education (ICFRE) - Arid Forest Research Institute (AFRI), Jodhpur, Rajasthan, India; ^3^ Department of Plant Breeding and Genetics, Odisha University of Agriculture and Technology, Bhubaneswar, Odisha, India; ^4^ Division of Agricultural Bio-informatics, Indian Council of Agricultural Research (ICAR)- Indian Agricultural Statistics Research Institute, New Delhi, India; ^5^ Division, Social Science Division, Indian Council of Agricultural Research (ICAR)- National Rice Research Institute (NRRI), Cuttack, Odisha, India; ^6^ Division of Computer Application and IT, National Institute for Agricultural Economics and Policy Research (NIAP), New Delhi, National Capital Territory of Delhi, India; ^7^ Department of Seed Science and Technology, Chaudhary Charan Singh Haryana Agricultural University, Hisar, Haryana, India; ^8^ Crop Protection Division, Indian Council of Agricultural Research (ICAR) - Vivekananda Parvatiya Krishi Anusandhan Sansthan, Almora, Uttarakhand, India; ^9^ Division of Entomology, Indian Council of Agricultural Research (ICAR-IARI)- Indian Agricultural Research Institute, New Delhi, India; ^10^ Department of Entomology, Maharana Pratap University of Agriculture and Technology, Udaipur, Rajasthan, India; ^11^ Division of Plant Pathology, Indian Council of Agricultural Research (ICAR)- Indian Agricultural Research Institute, New Delhi, India; ^12^ Department of Plant Breeding and Genetics, Assam Agricultural University, Jorhat, Assam, India; ^13^ Department of Entomology, Dr. Rajendra Prasad Central Agricultural University, Samastipur, Bihar, India; ^14^ Department of Entomology, Navsari Agricultural University, Navsari, Gujarat, India; ^15^ Department of Biology, College of Science and Humanities in Al-Kharj, Prince Sattam Bin Abdulaziz University, Al Kharj, Saudi Arabia; ^16^ Department of Biology, College of Science and Humanities, Prince Sattam Bin Abdulaziz University, Al Kharj, Saudi Arabia

**Keywords:** horticultural crops, endophytic entomo-pathogenic fungi (EEPF), insects, biological control, EEPF- plant colonisation

## Abstract

Horticultural production is a vital catalyst for economic growth, yet insect infestations reduce horticultural crop yield and quality. Pesticides and other pest control methods are used during planting to eliminate pests that cause direct and indirect losses. In such situations, endophytic entomo-pathogenic fungi (EEPF) can act as a potential tools for biological control. They protect plants by boosting growth, nutrition, morpho-physiology and salt or iron tolerance. Antixenosis, antibiosis and plant tolerance change insect performance and preferences. EEPF- plant colonisation slows herbivore development, food consumption, oviposition and larval survival. EEPF changes plant physio-chemical properties like volatile emission profile and secondary metabolite production to regulate insect pest defences. EEPF produces chitinases, laccases, amylases, and cellulases for plant defence. Recent studies focused on EEPF species’ significance, isolation, identification and field application. Realizing their full potential is difficult due to insufficient mass production, storage stability and formulation. Genetic-molecular and bioinformatics can help to build EEPF-based biological control systems. Metagenomics helps study microbial EEPF taxonomy and function. Multi-omics and system biology can decode EEPF interactions with host plants and microorganisms. NGS (Next Generation Sequencing), comparative genomics, proteomics, transcriptomics, metabolomics, metatranscriptomics and microarrays are used to evaluate plant-EEPF relationships. IPM requires understanding the abiotic and biotic elements that influence plant-EEPF interaction and the physiological mechanisms of EEPF colonisation. Due to restricted research, there are hundreds of unexplored EEPFs, providing an urgent need to uncover and analyse them.

## 1 Introduction

The current global population is estimated to be around 7.7 billion people, with a projected increase to 10 billion by 2050 (Etesami and Jeong, 2018). Population growth has a significant impact on the environment and climate change caused by human activities poses a serious threat to the food supply and people’s livelihoods (Food and Agriculture Organization, 2008; United Nations, 2019). With limited resources, the agriculture sector is struggling to feed such a large population. Intensive agriculture, defined by increased pesticide and fertiliser use and a lack of crop diversification poses a serious threat to biodiversity and ecological processes (Flynn et al., 2009). Although the global food system is more reliant on a few staple cereals, which account for roughly 60% of plant-based human energy intake, there is enormous potential to include horticultural produce to diversify diets and improve human health (Tiwari and Charuvi, 2021). The worldwide horticulture market is anticipated to be valued at USD 20.77 billion in 2021, rising at a CAGR of 10.2% to USD 40.24 billion by 2026 (GME, 2022). A variety of abiotic and biotic variables, however, have a detrimental effect on horticultural crop yield. Drought, salt, heat, and cold are the main abiotic stressors affecting horticulture crops and they can cause losses of up to 50 -70% (Tiwari et al., 2020), whereas biotic stresses, primarily insects and diseases, can cause losses of 40-60% (Oerke and Dehne, 2004). Amongst all biotic constraints, insect pests are one of the major concerns across the globe (Saha et al., 2020). Horticultural crop growers are most concerned with developing an eco-friendly management method that is compatible with the current pest control strategy, given the risks associated with overusing chemicals, such as pest resurgence, resistance, pesticide residue and biomagnification at higher trophic levels (Banjo et al., 2003). Entomopathogenic microorganisms are a non-chemical, sustainable pest management solution (Fanning et al., 2018). Entomopathogenic fungi (EPF) have cost-effectiveness, increased output potential, no pesticide residues, and enhanced biodiversity (Litwin et al., 2020). However, only a few genera, including *Lecanicillium*, *Metarhizium*, *Hirsutella, Isaria* and *Beauveria* have been commercialized as entomopathogens. Thus far, 12 species of Oomycetes, 65 species of Chytridiomycota, 339 species of Microsporidia, 474 species of Entomophtoromycota, 238 species of Basidiomycota, and 476 species of Ascomycota have been reported (Litwin et al., 2020). To date, more than 700 species from approximately 90 different genera have been established as insect-pathogenic fungi (important EPFs have enlisted in **Table 1**) (Khachatourians and Qazi, 2008), while, only 170 strains have been formulated as mycopesticides and are available for commercial use (Bamisile et al., 2021). *Metarhizium, Beauveria, Paecilomyces, Isaria* and *Lecanicillium*-based biopesticides have all received widespread application (Chen et al., 2015). When it comes to controlling pests and fungal plant pathogens, *Beauveria bassiana* has the highest endophytic capacity among EPFs in roughly 25 plant species (Vega, 2018). While *I. fumosorosea* does not appear to have any major plant interactions. Although *Isaria* spp. are known to be susceptible to plant defence chemicals, *I. fumosorosea* isolates have been reported to be effective against root nematodes, *Meloidogyne javanica* despite very low infection rates (Inglis et al., 2001; Zimmermann, 2008). EPFs are highly host specific, UV instability and lesser ecological tolerance hinder their wider applicability. Keeping these in view, inhabiting/incorporating these fungi within plants could lead to noble ways to modify fungal entomopathogens’ interactions with plants (Cory and Ericsson, 2010). In addition to direct insect pest control, numerous fungal entomopathogens were documented to have within plant development history (Schulz and Boyle, 2005) as naturally existing endophytes and various successful attempts have been made to intentionally incorporate EPFs into plants (Vega, 2018). Various plant qualitative and quantitative characters were reported to be enhanced through the natural or artificial colonization of EPFs in plants (Klieber and Reineke, 2016). As a result, another possible strategy for combating horticultural crop pests could be to inoculate plants with highly virulent endophytic entomopathogenic fungi (EEPFs) (Arnold and Lewis, 2005). EEPFs colonise plants systemically, providing sustained resistance against insect pests and fungal entomopathogens. Due to a lack of research, many important EEPFs may remain operational but undetected. Some endophytes may become extinct due to pollution, habitat fragmentation, biodiversity loss, and deforestation (Kandalepas et al., 2015). These biological agent reservoirs should be researched for pest and disease management and to understand their variety and functionality in various agro-ecological zones. The current study focuses on the modus operandi of EEPF-plant association with insects, consequences and factors influencing the EEPF-tritrophic continuum, artificial inoculation of EEPFs in plant systems, and molecular approaches for unravelling the beneficial coordination, which can be used in plant protection measures.

**Table 1:** List of Entomopathogenic Fungi (EPF).

SL. No.	Division of entomopathogenic fungi	Examples	References
1.	Ascomycota	*Metarhizium (M. anisopliae, M. robertsii, M. brunneum,M. lepidiotae, M. globosum, M. acridum, M. majus, M. flavoviride, M. rileyi, M. pingshaense, M. lepidiotae and M. guizhouense*)	Tkaczuk et al., 2015; Jaihan et al., 2016
		*Beauveria (B. bassiana* and *B. brongniartii)*	
		*Isaria* (*I. fumosorosea-formerly Paecilomyces fumosoroseus, I. farinosa and I. tennuipes*)	
		Ophiocordyceps (*O. sinensis*-formerly *Cordyceps sinensis, O. unilateralis*)	
		Cordyceps (*C. militaris*), Torubiella (*T. ratticaudata*)	
		Pochonia (*P. chlamydosporia*)	
		Hirsutella (*H. thompsonii, H. nodulosa, H. aphidis*)	
		Lecanicillium (*L. lecani*-formerly *Verticillium lecanii, L. longisporum*)	
2.	Entomophtoromycota	*Furia, Conidiobolus, Entomophaga*, or *Erynia*	Litwin et al., 2020

## 2 Fungal entomopathogens as endophytes

Endophytes are widespread in the plant kingdom, forming connections with a variety of organisms and providing secondary protection against pests (Hartley and Gange, 2009). Endophytic fungi are plant-associated microorganisms that colonise and live within a plant viz., roots, stems or leaves (Suryanarayanan, 2013) without adversely affecting it (Puri et al., 2016) and they can be either mutually beneficial root endophytes or plant-related endophytes (Vega, 2008). The relationship of endophytic fungi with higher vascular plants (Arnold and Lewis, 2005) is referred to be symbiotic because the former assists their hosts return for nutrients from the hosts and in return it provides indirect protection against herbivores (Quesada-Moraga et al., 2009). By infecting sucking pest complexes, lepidopteran larvae, and other cosmopolitan insects, fungal endophytes have the potential to function as bio-agents that cause disease in insects. It has been established that they can infect specific hosts while posing a risk that is negligible or nonexistent to other species, including those that are beneficial (Akutse et al., 2014). For example, *Beauveria bassiana*, has been identified as an endophyte in tomato (Ownley et al., 2004), cocoa (Evans et al., 2003), potato (Jones, 1994), date palm (Gómez-Vidal et al. 2009), bananas (Posada et al., 2007) and opium poppy (Quesada-Moraga et al., 2006). Successful inoculation of cocoa and coffee seedlings with *B. bassiana* has been achieved by applying a spore suspension to the radicle immediately after germination. In addition, inoculation of the the fungi *Metarhizium brunneum* in *Capsicum annum* (sweet pepper) (Jaber and Araj, 2018) and *Paecilomyces* sp. in *Musa acuminata* were also reported to be successful (Cao et al., 2002).

## 3 Mechanism of successful colonization through EEPF offense vs. plant defense

**Figure 1:**
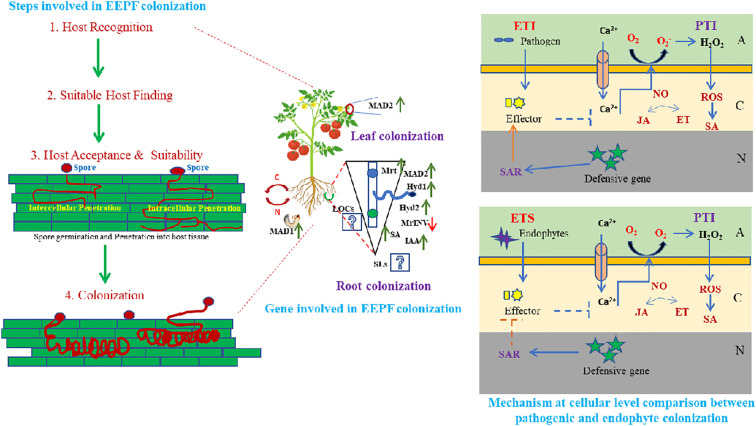
Host identification and establishment by entomopathogenic fungal endophytes involves diverse steps i.e. host recognition, suitable host finding, host acceptance and suitability followed by spore germination and penetration into host tissue leading to fungal colonization. This colonisation can occur in any of the plant parts preferably roots and leaves either intra or intercellularly, leading to diverse local and/or systemic biochemical and molecular genetic up-regulation. Various adhesion molecules, MAD1 in insects and MAD2 in *Metarrhizium anisopliae* and hydrophobins (Hyd1, Hyd2) in *Beauveria bassiana*, Mrt (*Metarrhizium* raffinose transporter), MrlNV (extracellular invertase in *Metarrhizium*), certain fungal derived plant hormone like IAA (Indole acetic acid) and SA (Salicylic acid) are considered to be essential for spore adhesion. While, certain LCOs (Lipochitooligosaccharides) and SLs (Strigolactones) promote EEPF- plant colonisation as signalling molecules, that initiates a chain of systemic reaction in plants, leading to EEPFs establishment in plants. Plant cell wall acts as the first line of defense, overcoming which EEPFs gain access to the innate immune system of plants after the MAMP (Microbe associated molecular pattern) or PAMP (Pathogen associated molecular pattern) is recognised by the plants, leading to activation of MTI (MAMP- triggered immunity) or PTI (Pattern triggered immunity). Further, plants secrete certain effector molecules, that leads to downstream synthesis of SA (Salicylic acid), JA (Jasmonic acid), NO (Nitric oxide) and generation of ROS (Reactive oxygen species), which enables the EPF (Entomopathogenic fungi) to overcome the ETI (Effector triggered immunity) (essential for pathogenesis) and making the plant susceptible for EEPF colonisation through ETS (Effector triggered susceptibility) (essential for endophytism). (A, Apoplast; C, Cytoplasm; N, Nucleus).

The EEPF infection process begins with conidia, the fruiting body, which transforms into a germ tube after penetrating the host plant and becoming hypha (Dash et al., 2018). In both plants and insects, the infection process continues after entry into the host tissue (via natural openings of epidermal cells/mechanical pressure). The plant and the EEPF must first overcome a variety of environmental stresses that are critical for plant growth and development (Manoussopoulos et al., 2019). For EEPF-insect interactions, the genera *Beauveria* and *Metarhizium* have been reported to serve as general models (Moonjely et al., 2016), and these EEPFs follow similar modus operandi to enter and establish inside their plant and insect hosts, with similar genetic involvement, which could have resulted from gene duplication or horizontal gene transfer (Zhang et al., 2019), indicating co-evolved processes (Moonjely et al., 2016). Invading insects search for suitable plant hosts, recognise hosts through associated molecular patterns, accept and suitability mediated by asexual spore attachment to host surfaces and invade and multiply inside plant tissue, where the EEPF establishes itself after evading the insect’s immune system (Vega et al., 2012). Through its colonisation of plant tissues and infection of insects, EEPF forms a tripartite interaction in which it, the plant and the insect share nutrients (**Figure 1**). Most of what is known about these connections come from the fungus *Metarhizium robertsii*, whose mycelium colonises root cells and soil-dwelling larvae (Behie et al., 2012). The results of sophisticated radioactive isotope studies suggested that *M. robertsii* absorb carbon from their host plant and transfer nitrogen from insects to their root systems (Behie et al., 2017). These studies monitored ^15^N and ^13^C in *M. robertsii* and plants and discovered that the fungus takes carbon from the plants and uses it to make chitin and trehalose, two types of fungal carbohydrates. As a result, we can hypothesise that EEPF, plants and insects engage in intricate tritrophic interactions. Different adhesion molecules, including MAD1 in *M. robertsii*, and hydrophobins in *Beauveria*, were reported to be required for spore adhesion to the insect cuticle (Zhang et al., 2011). Furthermore, certain surface proteins recognised insect cuticular proteins and initiated cuticular degradation. Following successful adherence, the conidia form hyphae, which then differentiate into blastospores in the insect haemocel, which produces beauvericin and destruxins, which are insecticidal metabolites that cause insect mortality (Barelli et al., 2016). In addition, EEPFs produce antimicrobial compounds in the dead cadaver to reduce microbial competition and ensure adequate nutrient allocation (Fan et al., 2017). In contrast to the establishment of EEPFs in insects, the EEPF-plant adhesion process is dependent on the adhesin MAD2, which is similar to MAD1 in insect adhesion (Behie et al., 2015). *Metarhizium* adhesion to plant epidermis was impaired by genetic deletion of MAD2 (Wang and Leger, 2007). Proteins such as Hyd1 (Hydrophobin1), Hyd2 (Hydrophobin2), Mrt (*Metarhizium* raffinose transporter), MrINV (extracellular invertase in *Metarhizium*), fungal-derived plant hormone (IAA), and plant hormone (SA). Furthermore, different EEPF LCOs (lipochitooligosaccharides) and plant SLs (strigolactones) act as signalling metabolites in plants - EEPF colonisation (Hu and Bidochka, 2021). After overcoming the first line of defence, the recognition of microbe or pathogen-associated molecular patterns (MAMPs or PAMPs) like conserved molecules and activating MAMP-triggered immunity (MTI) and/or pattern triggered immunity (PTI) is carried out by pattern recognition receptors (PRRs) present in plant cells (Newman et al., 2013); while plants secrete certain effector molecules, which induce effector triggered immunity (ETI) against the potential pathogen (Mendoza-Mendoza et al., 2018). A growing body of research suggests that beneficial microbes may evade ETI or short-circuit plant defence responses at the effector-triggered susceptibility state to allow successful colonisation of host roots or by evading recognition by removing or mutating recognised effectors (Zamioudis and Pieterse, 2012). Another notable example of PTI is the EEPF chitosan molecules, which have been shown to induce host defence (Sánchez-Vallet et al., 2015). LysM is a carbohydrate-binding module found in many extracellular proteins and receptors that recognise polysaccharides containing N-acetylglucosamine residues (in EEPF chitosan). Chitin and chitin polymers, as well as their modified form, chitosan, have been shown to induce host defence responses in a variety of horticultural crops. In the context of fungal endophytes, plant chitin-specific receptors (PR-3) recognise chitin oligomers produced on the fungal cell wall, which accelerates subsequent defensive reactions (Sánchez-Vallet et al., 2015). In response to plant defensive behaviour, EEPFs have been shown to elicit counteractive features such as molecule secretion for non-recognition of chitin, defensive enzymes and production of salicylate hydrolase to inhibit salicylic acid defence and a raffinose transporter and an extracellular invertase for sucrose hydrolysis in plant roots. EEPFs, on the other hand, devise mechanisms to protect themselves from plant defence mechanisms. Chitin deacetylases, for example, deacetylate chitosan oligomers, rendering them unrecognisable by plant receptors (Cord-Landwehr et al., 2016). In addition, certain defensive enzymes such as catalases (CAT), superoxide dismutases (SOD), alkyl hydroperoxide reductases (AhpC), peroxidases (POD) and glutathione-S-transferases (GSTs) are activated in response to oxidative burst (Zeidler et al., 2004). Although the details on all of the genes involved in EEPF-plant establishment are unknown, research on *M. robertsii* has highlighted the plant colonisation and endophytism mechanisms. The salicylic acid (SA) cascade is started by the plant’s immune system and is dependent on the rate at which spores adhere to and penetrate the plant (Martínez-Medina et al., 2017). Low spore adherence can lead to decreased root tissue penetration, which may allow *B. bassiana* to generate stronger endophytes. An examination of the activation of the immune system in plants in response to *B. bassiana* colonisation shows that it can overcome SA-dependent systemic acquired resistance by producing a (fungal) salicylate hydrolase enzyme. The fungus can grow endophytically without affecting the plant’s immune system. According to a study, *M. robertsii*’s establishment in plants depends on a raffinose transporter and a sucrose-hydrolyzing extracellular invertase (Fan et al., 2017). Root exudates contain raffinose and sucrose, which are required for *M. robertsii* rhizosphere proliferation and root competence. Plant offence and EEPF defence boost colonisation by making raffinose and sucrose available for EEPF growth and colonisation. EEPFs could provide multimodal protection for plants against phytophagous insects, either directly (e.g., poisonous chemical production) or indirectly [volatile organic compounds (VOCs)] to attract natural enemies. Understanding the tritrophic interaction involving EEPF- colonised plants is thus required, as described below.

## 4 Consequences of multitropic interactions of EEPF in the trophic chain

Plant-associated microorganisms help plants, herbivores, and natural enemies interact. Endophytes can change host resistance to herbivores and natural enemies (Hatcher, 1995). Systemic or transitory endophytic colonisation provides multimodal protection to plants by affecting multitrophic relationships between pest species and plants, as well as between insect pests and their predators, parasitoids, and natural enemies (Quesada Moraga, 2020). Endophytes impact and modulate plant metabolic processes because they live in and interact with plants. In some cases, the benefitting plant and endophyte share a pathway to produce new compounds (Poling et al., 2008). Both partners can modify metabolite backbones to create new metabolites (Pimentel et al., 2011). The effects and consequences of EEPF colonisation at various trophic levels, as well as their interaction, are described below with appropriate examples.

### 4.1 Trophic level one: EEPF and plant

With the gradual exposure to a wide range of positive effects imparted by endophytic fungi on their hosts, the close interaction between plants and endophytes has gotten a lot of attention. Fungal endophytes help to boost plant robustness (Khan et al., 2012), plant growth, nutrient intake capacity, photosynthesis, and plant hormone levels (Li et al., 2018). Recent studies have demonstrated that EEPFs have additive effects on host plant growth and development by enhancing plant height, plant weight and growth rate (Greenfield et al., 2016; Jaber and Araj, 2018). The insect derived nitrogen is transferred from endophytic *Metarhizium* spp. to the host plant and in turn it receives carbon from the host plant (Behie and Bidochka, 2014; Behie et al., 2017). Furthermore, the fresh weight of roots, shoots and the height of broard bean, *Vicia faba* were reported to increase after foliar treatment with EPF, *B. bassiana, B. brongniartii*, and *M. brunneum* (Jaber and Enkerli, 2017). Similiarly, tomato plants treated with *M. anisopliae* were reported to boost plant height, root length, shoot as well as root dry weight (Elena et al., 2011). Furthermore, soil drenching with fungal conidial suspension resulted in enhanced growth of sweet pepper (*Capsicum annum*) (Jaber and Araj, 2018). Numerous abiotic and biotic factors such as plant and microbe genotypes, climatic circumstances, the dynamics of interaction within the plant and soil biomes and the inoculation method were reported to influence the extent of endophytism (Raya-Díaz et al., 2017). Furthermore, plants are able to withstand biotic stresses (underground herbivory by nematodes, root-feeding insects) and abiotic stresses (salt, drought, heat stress) because EEPFs have been shown to release toxins that impede the development and proliferation of other competitors, including pathogenic organisms (Clark et al., 1989; Khan et al., 2012; Cosme et al., 2016).

### 4.2 Trophic level two: EEPF and herbivore

Numerous studies have demonstrated that endophytic entomopathogenic fungi can inhibit insect pests that feed on microbe-colonized plants (Sánchez-Rodríguez et al., 2018). Endophytic fungi reduce insect herbivore damage by inhibiting insect development (Akutse et al., 2013), providing a feeding deterrent (Vega, 2008), retarding insect development and restricting insect survival and oviposition (Martinuz et al., 2012). According to Leckie (2002), the incidence of *Helicoverpa zea* was reported to be reduced in *B. bassiana* treated tomato plants. Klieber and Reineke (2016) observed 50% mortality and reduced longevity in the larvae of *Tuta absoluta* (Meyrick) on *B. bassiana*-treated tomato leaves. Moreover, Qayyum et al. (2015) found a reduction in *H. armigera* damage in tomato plants in response to *B. bassiana* colonisation. Similar results were also obtained by Posada et al. (2007) when the coffee berry borer (*Hypothenemus hampei*) was fed on *B. bassiana*-treated coffee plants. Furthermore, Akello et al. (2008) showed a comparable decline in the larval survival and overall population of the banana weevil, *Cosmopolites sordidus* (Coleoptera: Curculionidae). The bulk of these studies link insect pest damage reduction due to mycotoxin accumulation in plant tissues (Gurulingappa et al., 2011). Lozano-Soria et al. (2020) revealed that volatile compounds produced by two EEPFs, *B. bassiana* (Bb1TS11) and *M. robertsii* (Mr4TS04), can deter the banana Rhizome weevil, *C. sordidus*. Gas chromatography/mass spectrometry-solid- phase micro extraction (GC/MS-SPME) studies revealed the identification of 97 diverse VOCs, out of which seven VOCs (styrene, benzothiazole, camphor, borneol, 1,3-dimethoxy-benzene, 1-octen-3-ol and 3-cyclohepten-1-one) were found to have insect repellent activity (Lozano-Soria et al., 2020). *B.bassiana* has also been shown to have insect repellent properties against the palm weevil, *Rhynchophorus ferrugineus*. Whitefly, *Bemisia tabaci* does not prefer *B. bassiana* inoculated tomato seedlings, according to Wei et al. (2020), because of the formation of bioactive chemicals chitin and glucans. Destruxin A was discovered to be effective against whiteflies 48-72 hours after *Metarhizium brunneum* colonisation in diverse tissues of potato plants and melon leaves (Garrido-Jurado et al., 2017). However, not all fungi can colonise plants due to their inability to adapt to the plant’s nutrients (Mercado-Blanco and Lugtenberg, 2014). Transient fungal/plant interactions may remain for several days after spraying due to the broad distribution of leaves (Mantzoukas and Lagogiannis, 2019). According to recent studies, EPF’s temporary endophytic colonisation of plant tissues kills chewing and sucking insects that feed exophytically on plants (Resquín-Romero et al., 2016).

### 4.3 Trophic level three: EEPF and natural enemy

The complete understanding of herbivores and EEPF colonized host plants require integration of the third trophic level (Price et al., 1980). The slow growth-high mortality hypothesis (SG-HM) revealed that the altered nutri-allelochemistry of the EEPF associated host plants prolonged the larval development that ultimately enhances the qualitative and quantitative foraging efficiency of predators and parasites (Clancy and Price, 1987). The multitrophic relationships among *M. brunneum* colonized host plants - pest *Spodoptera littoralis* (Boisduval) (Lepidoptera: Noctuidae)- natural enemy *Hyposoter didymator* indicated 33% higher parasitisation and reduced reproductive potential of the parasitoid on *Spodoptera littoralis* fed on *M. brunneum* inoculated host plants than on non-inoculated ones (Miranda-Fuentes et al., 2020). Moreover, the rate and time of *Aphis gossypii* (prey) consumption by *Chrysoperla carnea* and rate of mummification by *Aphidius colemani* was not significantly differed in response to endophytic colonisation by *B. bassiana* (González-Mas et al., 2019a). However, predator behaviour on aphids infesting *B. bassiana* endophytically colonised plants concluded the potential use of EEPFs in IPM programmes in combination with other biocontrol agents that would provide additive pest suppression (Gonzalez-Más et al., 2019b). EEPFs are safe and compatible with other biocontrol agents, but this less explored area requires a better understanding of potential changes in the chemical ecology of the plant post-colonization, multitrophic relationships between insects and plants, and insects and their entomophagous natural enemies. Endophytic colonisation of plants can modify volatile content, resulting in differential biotic and/or abiotic tolerance/resistance. When parasitoids and predators work with EEPFs, insect herbivores and plant damage are minimised. Jaber and Araj (2018) suppressed the green peach aphid (*Myzus persicae* Sulzer) with EEPF *B. bassiana*, *M. brunneum* and *Aphidius colemani* Viereck. Prior research suggests that EEPFs negatively influence natural enemies’ development, reproduction, and adult survival (Omacini et al., 2001; Kunkel et al., 2004). Mycotoxins travel from colonised plants to insects and ultimately to their parasites. Kunkel et al. (2004) claimed that endophyte-produced poisons could cause indirect harm to natural adversaries (Omacini et al., 2001). Insect herbivores that consume EEPF-colonized plants may be smaller and have less nourishment (Richmond et al., 2004).

### 4.4 Effect of EEPF colonised plants on insect-natural enemy interaction through production of secondary metabolites

Aside from directly influencing tritrophic interactions, certain volatile organic compounds (VOCs) and secondary metabolites produced by EEPF-colonized plants have significant effects. Sphaeropsidin A (SphA) is a pimarane diterpene that has been shown to have larvicidal and phagodeterrent effects on lepidopteran insects such as *Spodoptera littoralis* (Andolfi et al., 2014). SphA was discovered to have contact and oral toxicity against the chewing lepidopteran *S. littoralis*, depending on its ability to perform SphA biosynthesis *in vivo* (Di Lelio et al., 2022). An *in-vitro* study reveals the sub-lethal effect of a partially-purified protein derived from the EEPF, *Lecanicillium lecanii* (Zimmerman), associated with solanaceous crops such as tomato, on the green peach aphid *Myzus persicae* (Sulzer). Furthermore, RT-qPCR expression analyses of key genes associated with the salicylic acid (SA) and jasmonic acid (JA) pathways were found to be upregulated and thus revealed significant negative effects on *M. persicae* survival and fecundity. The *L. lecanii*-derived protein was found to strongly enhance the SA associated genes PR1, BGL2, AOS, PAL, LOX and AOC, indicating the enhancement of systemic resistance in plants, implying that it should be purified and characterised as a novel biomolecule against aphids and other phloem-feeding insect pests (Hanan et al., 2020). *Trichoderma harzianum* Tr6 is also a potent inducer of induced systemic resistance (ISR) and can stimulate the immune system in plants and also adversely affect the host preference and egg production of *Trialeurodes vaporariorum* (Aldaghi et al., 2021). Furthermore, the systemic effects of EEPFs (*Trichoderma asperellum, Gibberella moniliformis, B. bassiana, M. anisioplaie*, and *Hypocrea lixi*) application on *Vicia faba via* seed treatment were observed and the aphid growth rate, offspring performance and fecundity were found to be retarded. Endophyte treatment was reported to reduce the number of *Aphis fabae* and *Aphis pisum* nymphs by 1.6-14.6 and 3.7-11.0 times, respectively, while endophyte seed treatment increased seedling survival by 20-100% compared to none in the control treatment at 20 days post infestation (Akello and Sikora, 2012). Individual or combined inoculation of *Rhizophagus intraradices*, an arbuscular mycorrhizal fungus and *B. bassiana*, an EEPF, resulted in increased levels of monoterpenes and sesquiterpenes in beet armyworm (*Spodoptera exigua* Hübner) - infected tomato plants, indicating a stronger terpenoid mediated defence response in host plants (Shrivastava et al., 2015). Four *Trichoderma* isolates, including two trichodiene (TD) (a non-phytotoxic VOC) producers (T34-5.27, E20-5.7) and their parental strains (T34, E20) were evaluated, and differential host preference (higher repellancy with the E20 strain) and altered emergence (reduced emergence with the E20 and T34 strains) of *Acanthoscelides obtectus* were observed in wild and cultivated common beans colonized by *Trichoderma* isolates (Rodríguez-González et al., 2019).

### 4.5 Impact of climate change on EEPF and its interaction with tropic levels

Climate change affects many biological processes and may impact bottom-up and top-down agroecosystem characteristics through tritrophic interactions (Chidawanyika et al., 2019). Global warming can alter interspecies relationships and community structure (Boukal et al., 2019) and thus influence the variety, distribution and function of plant associated microorganisms (Cavicchioli et al., 2019). Plants interact selectively with their microbiota to upregulate abitic, biotic stress tolerance and growth enhancement (Rodriguez and Durán, 2020). Temperature impacts plants’ physiology, chemistry, life cycle stages, development and growth, which affects microorganism-plant interactions (Pieterse and Dicke, 2007). Sui et al., 2020 explored temperature - EEPF interactions and temperature-induced physiological changes (2020) in *Zea mays* and *Beauveria bassiana*. In the ambient temperature range, maize’s photosynthesis and respiration were reported to be increased (Bokhorst et al., 2010); PAL (Phenyl Alanine Ammonia Lyase) and PPO (Polyphenol Oxidase) are overexpressed in stress-adapted cells. Although *B. bassiana* develops at 20^0^ to 30^0^C, EEPFs develop normally at an elevation of 2^0^ C (Rangel et al., 2010). Under elevated air temperature, maize had positive effects on *B. bassiana* (conidia yield, germination rate of conidia and virulence), while *B. bassiana’s* growth and biological characteristics remained unchanged. This suggests that elevated air temperature could shift the interactions between plants and EEPFs, possibly from mutualism to commensalism. Moreover, EEPFs’ rapid adaptation and ability to ‘transfer’ resistance to their hosts may speed plants’ climate change adaption, which can be regarded as positive feedback and better exploited for breeding varieties with higher colonisation potential of EEPFs to tolerate, adapt and mitigate climatic stresses. EEPFs may accelerate climate change responses in crops and wild plant communities and require efforts to improve EEPF-facilitated plant health. Such information could help develop better climate change mitigation methods for plant communities (Suryanarayanan and Shaanker, 2021).

## 5 Constraints associated with EEPF colonization in plants and their artificial inoculation

**Figure 2:**
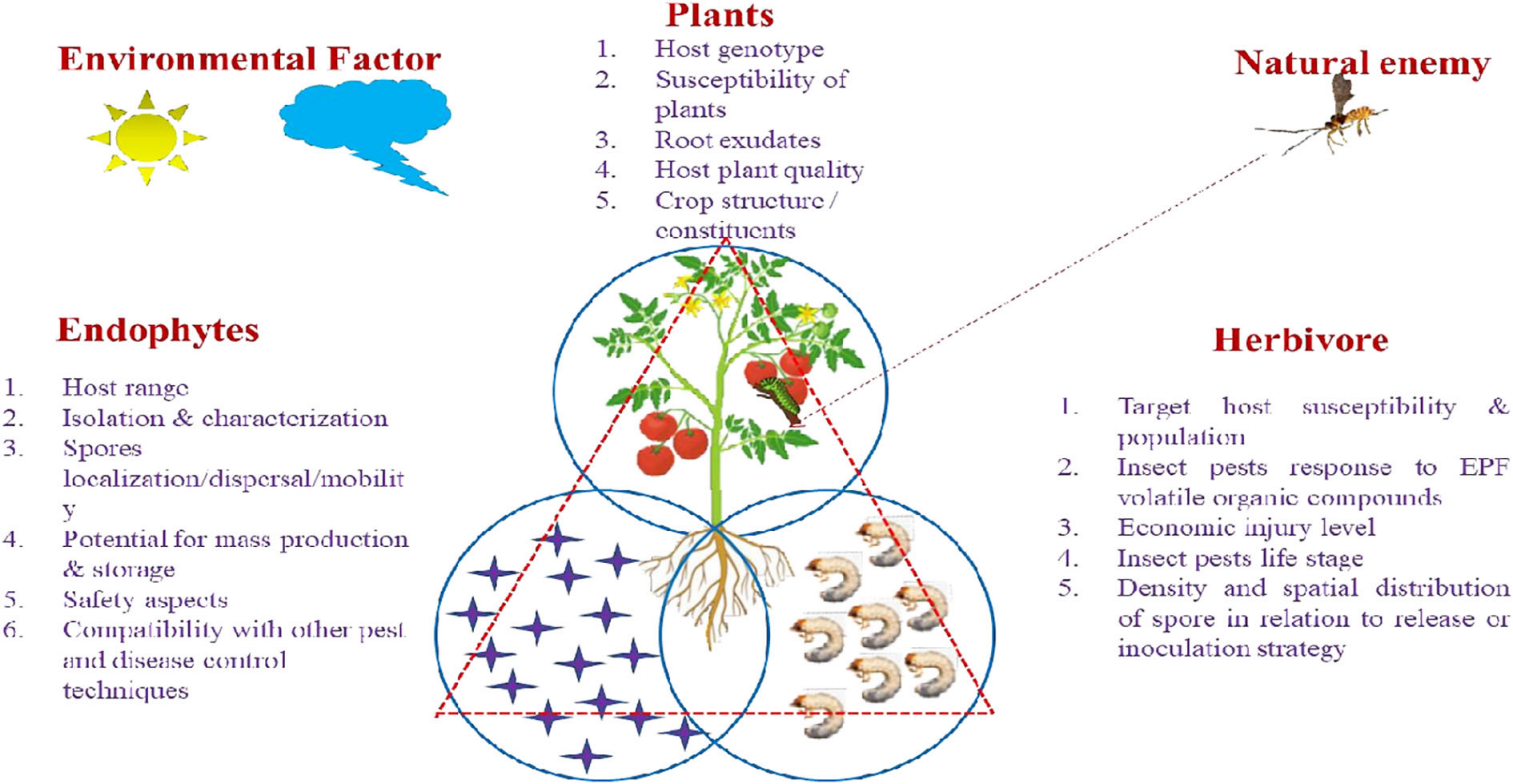
Specific Factors affecting EEPF- plant –insect interactions. The tritrophic interactions involving EEPF-colonised host plants-insects-natural enemies are dependent on diverse biotic factors (fungal features of EEPFs, plants, the associated herbivores) and abiotic factors (environmental factors). Biotic factors: 1. Fungal features: include epizootic potential (Spores germination, Sporulation and Virulence), persistence, host range, isolation and characterization, spores localization/dispersal/mobility, potentials for mass production, suitability for storage and formulation, fungal toxicological and safety aspects, compatibility with other pest and disease control techniques. 2. Host features: Target host susceptibility & population, insect pests response to EPF volatile organic compounds, economic injury level, insect pests life stage, density and spatial distribution of spore in relation to release or inoculation strategy. Abiotic factors: 1. Environmental factors: Relative humidity (RH), temperature, soil moisture and pH, rain, irrigation, dew drops, solar ultraviolet radiation (UV).

**Figure 3:**
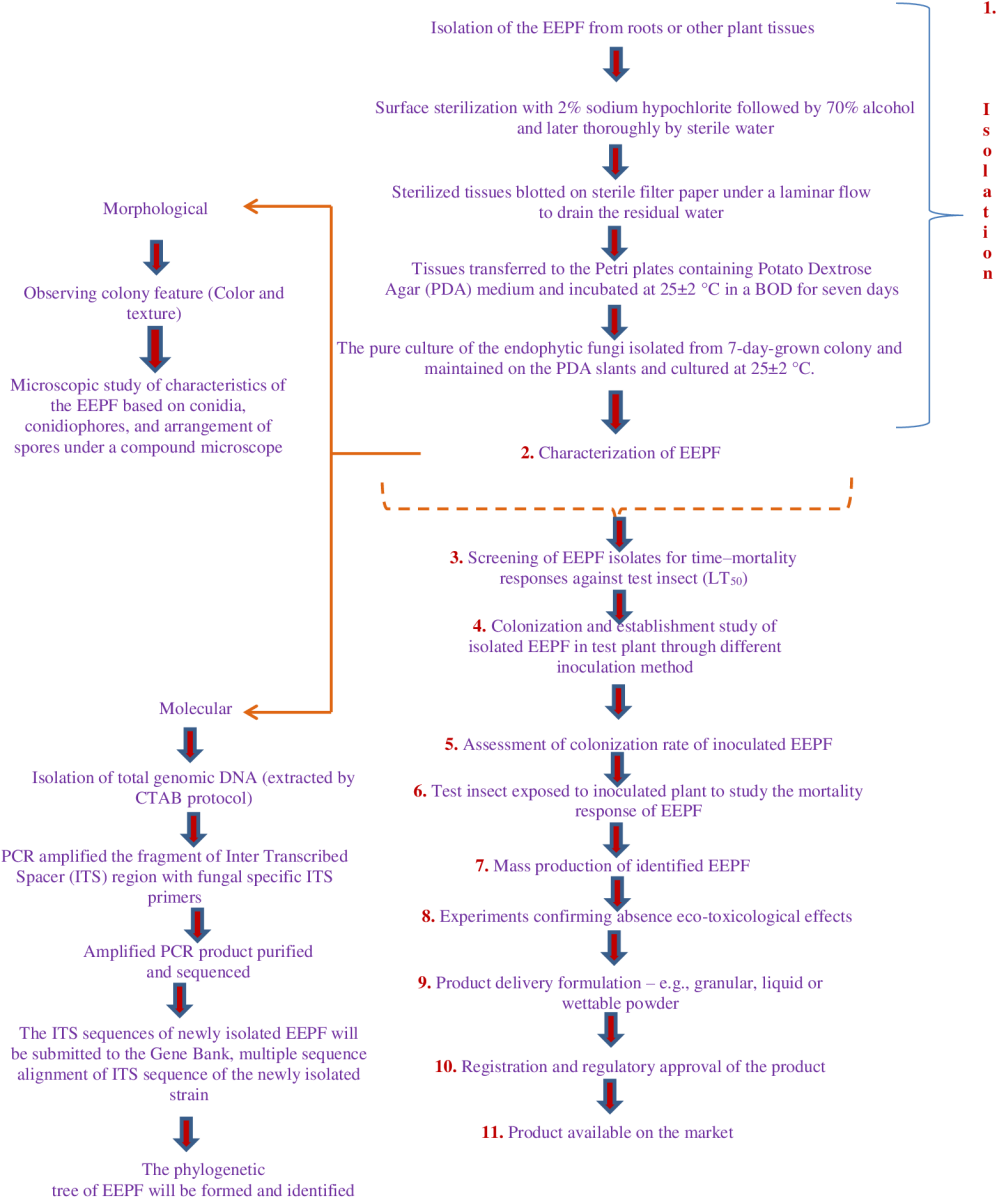
In recent years, EEPF-based plant protection strategies have received more attention. Current applications of these fungi do not support tailored formulations that would improve fungal colonisation near plant tissue and cause endophytism. Low fungal propagule stability during drying and storage, complex handling and high dosages and pricing per hectare are drawbacks. Formulation can improve all of these properties, bringing us closer to employing these EEPFs in IPM. Therefore, their isolation, identification, colonization, recovery, mass multiplication and commercialisation is utmost concern and the following steps can be adopted to develop commercialization roadmap of EEPF.

Weather patterns and soil conditions strongly influence the biology and ecology of fungi. Only one-tenth of the 1.5 million fungal species in soil have been examined. The natural relationship of EEPFs with plants is influenced by climate, vegetation, soil, location, and human and other biotic activities (Bing and Lewis, 1991) (Behie et al., 2014). However, other elements have been observed to play a role, such as an inoculum density, growing medium, plant age and species and fungal species (Parsa et al., 2013). Success in the endophyte colonisation of a host plant also depends on the isolate or isolates of EEPFs utilised, the intended crop species, and the growth environment (Qayyum et al., 2015) (**Figure 2**). However, a different study indicated that the growth media employed had a bigger impact on the plant’s endophytic colonisation than the inoculation strategy (Parsa et al., 2013). Thus, plants can be artificially inoculated with EEPFs using a variety of methods, such as foliar spraying, stomatal penetration, adhesion to the plant, seed dressing, soil inoculation, and even stem injections with varying results that could surpass the constraints associated with non-associated EEPFs in plants and thus affect the effectiveness of the fungal treatment and the degree of systemic colonisation (Vega et al., 2012). Colonisation can occur intracellularly, intercellularly, locally or systemically (Vega, 2018) or even vertically (Landa et al., 2013). **Table 2** contains a list of successful cases of artificial plant inoculation with endophytic EPF in horticultural crops. Because it is restricted and protected from both abiotic and biotic influences within the plant, endophytic EPF requires less inoculum than inundative applications to soil or substrate (Akello et al., 2008). Therefore EEPF’s isolation, identification, colonization, recovery, mass multiplication and commercialisation is of utmost concern in the present day. The potential roadmap for the commercialization of EEPF is detailed in flowchart form in **Figure 3**.

**Table 2:** Successful cases of artificial plant inoculation with endophytic EPF in horticultural crops against herbivore.

Sl. No.	Horticultural crop	EEPF	Inoculation method	Target pest	Country	Reference
1	Potato, *Solanum tuberosum*	*Beauveria bassiana*	Foliar application	European corn borer, *Ostrinia nubilalis*	North Carolina	Jones, 1994
2	sweet pepper, *Capsicum annuum*	*Metarhizium brunneum*	Soil denching	Green peach aphid, *Myzus persicae*	Jordan	Jaber and Araj, 2018
3	Grapevine, *Vitis vinifera*	*Beauveria bassiana*	Foliar application	Vine mealybug, *Planococcus ficus*	Germany	Rondot and Reineke, 2018
4	Banana, *Musa* spp.	*Beauveria bassiana*	Root and rhizome dip	Banana weevil, *Cosmopolites sordidus*	Uganda	Akello et al., 2008
5	Tomato, *Solanum lycopersicum*	*Beauveria bassiana*	Seed treatment	Corn earworm, *Helicoverpa zea*	USA	Powell et al., 2009
6	Tomato, *Solanum lycopersicum*	*Beauveria bassiana*	Root dip, injection, solid substrate and direct foliar application	Tomato shoot and fruit borer, *Helicoverpa armigera*	Pakisthan	Qayyum et al., 2015
7	Cauliflower, *Brassica oleracea*	*Beauveria bassiana, Metarhizium brunneum*	Foliar application	Sweetpotato whitefly, *Bemesia tabaci*	Jordan	Jaber et al., 2018
8	Strawberry, Fragaria × ananassa	*Beauveria bassiana, Isaria fumosorosea* and *Metarhizium anisopliae var. robertsii*	Rhizome treatment	Green peach aphid, *Myzus persicae*	Greece	Manoussopoulos et al., 2019
9	Pepper, *Capsicum annum*	*Beauveria bassiana, Metarhizium anisopliae* and *Isaria fumosorosea*	Foliar application	Green peach aphid, *Myzus persicae*	Greece	Mantzoukas and Lagogiannis, 2019
10	Common bean, *Phaseolus vulgaris*, Faba bean*, Vicia faba*	*Beauveria bassiana,Hypocrea lixii*	Seed soaking	Leaf minor, *Liriomyza* spp.	Kenya	Akutse et al., 2013
11	Onion, *Allium cepa*	*Clonostachys rosea, Fusarium sp., Hypocera lixi, Trichoderma harzianum*	Sedd treatment	Onion thrips, *Thrips tabaci*	Kenya	Muvea et al., 2014
12	Tomato, *Solanum lycopersicum*	*Acremonium strictum*	Soil drenching	American bollworm, *Helicoverpa armigera*	Germany	Jallow et al., 2008
13	Pumpkin, *Cucurbita maxima*	*Beauvaria bassiana, Lecanicillium lecanii, Aspergillus parasiticus*	Foliar spray	Melonor cotton aphid, *Aphis gossypii*, Australian plague locust, *Chortoicetes terminifera*	Austrelia	Gurulingappa et al., 2010
14	Cauliflower, *Brassica oleracea*	*Beauveria bassiana*	Foliar spray	Diamondback moth, *Plutella xylostella*	India	Gautam et al., 2016
15	Cucumber, *Cucumis sativus*	*Lecanicillium longisporum*	Foliar spray	Cotton or melon aphid, Aphis gossypii	Canada	Kim et al., 2010
16	Date palm, *Phoenix dactylifera*	*Beauveria bassiana*	Stem injection	Red palm weevil, *Rhynchophorus Ferrugineus*	Egypt	Arab and El-Deeb, 2012
17	Melon, *Cucumis melo*	*Beauveria bassiana and Metarhizium brunneum *	Foliar spray	Sweet potato whitefly, *Bemisia tabaci*	Spain	Garrido-Jurado et al., 2017
18	Melon, *Cucumis melo*	*Metarhizium brunneum*	Foliar spray	Leaf worm, *Spodoptera littoralis*	Spain	Miranda-Fuentes et al., 2021
19	Tomato, *Solanum lycopersicum*	*Beauveria bassiana*	Seed soaking, Leaf spraying, Root dipping	Tomato leafminer, *Tuta absoluta*	Argentina	Allegrucci et al., 2017
20	Faba bean, *Vicia faba*	*Beauveria bassiana*	Seed or leaf inoculation	Aphid, *Aphis fabae*	Denmark	Jensen et al., 2019

## 6 Integrated omics approaches to understand EEPF-host plant- insect interaction

The primary emphasis of these beneficial microbial associations was multitrophic interaction involving EEPF colonised plants-insects-natural enemies. However, the underlying physiological elements of endophyte-host interactions remain unknown. Identifying, isolating, and characterising the genes implicated in such beneficial connections is therefore crucial for efficiently regulating their interplay (Harith-Fadzilah et al., 2021). Plant-microbe interactions, particularly plant-EEPF interactions can be studied using multi-omics approaches. This thorough investigation of endophytic multi-omics data, from the genome to the metabolome, will aid in understanding their potential to biosynthesize secondary metabolites and lay the groundwork for the future development of this lucrative resource (Crandall et al., 2020). We explored the importance of several omics technologies in understanding the role of EEPFs and their interactions with related hosts in the following sections.

### 6.1 Genomics approaches

Owing to advancements in genome sequencing technology, identifying the fungus isolated from plants is now a cheap and quick process. One of the most popular programmes for sequence alignment, Basic Local Alignment Search Tool (BLAST), is utilised in the in-silico identification procedure to determine the degree of similarity between the sequences (Altschul et al., 1990). First, the coffee (*Coffea arabica*) fungal endophytes were identified using the BLAST approach and it was found that there were 15 different species in the area, two of which, *B. bassiana* and *Cladosporium rosea*, demonstrated pathogenicity against the coffee stem borer (Vega, 2008). The sequencing revolution and computer techniques for assembling and annotating genome sequences made fungal genome reconstruction conceivable. Many specialised metabolites are generated by pathways encoded by physically nearby genes on fungal endophyte genomes. These microbial secondary metabolites are a major untapped resource of natural products (NPs) with agrochemical and medicinal applications. Quantity and composition vary on host plant type, plant development phases, environmental stress, and other factors affecting plant growth, such as insect-pest attacks (Baudoin et al., 2003; Gunatilaka, 2006).

Therefore, utilizing gene clustering in EEPFs that are organized into operon structures under a single promotor, several computational methods have been developed to identify metabolic gene clusters and pursue the discovery of specialized metabolites. Co-inheritance, co-transcriptional regulation, and coordinated post-transcriptional processes, such as protein synthesis export, are benefits of gene clustering. Genome sequencing and annotation for *M. acridum* and *M. robertsii* (Gao et al., 2011), *B. bassiana* (Xiao et al., 2012), and *M. anisopliae* (Pattemore et al., 2014) give information on secondary metabolite encoding. With so much genetic data, secondary metabolite gene clusters are abundant. The present objective is to tie biosynthetic gene clusters (BGCs) to as many known molecules as feasible and forecast the molecules that encode the most promising compounds. NP.searcher (Chavali and Rhee, 2018), ClustScan (Starcevic et al., 2008), CLUSEAN (Weber et al., 2009), antiSMASH (Medema et al., 2011), SMURF (Khaldi et al., 2010), MIDDAS-M (Umemura et al., 2013) and ClusterFinder (Cimermancic et al., 2014) like computational approaches have helped researchers uncover genes producing secondary metabolites like Non-ribosomal Peptide Synthases (NRPSs), NRPS-like enzymes, and polyketide synthases (PKSs). Most core enzymes were distributed into 75 BGCs (Wang et al., 2015). To comprehend and manipulate EEPFs, and clarify endophytes’ metabolic potential and beneficial qualities (Dinsdale et al., 2008), metagenomics allows the discovery of new genes, proteins or even full genomes of uncultivable organisms in less time and with more precision than conventional microbiological and molecular approaches, providing information beyond individual taxon genomics. Amplicon sequencing and whole genome shotgun sequencing are used to study the microbiome. Internal Transcribed Spacers (ITS) of nuclear ribosomal DNA (rDNA) serve as a marker to distinguish most fungal species since it is extremely repetitive and variable portions are flanked by more conserved DNA sequences (Hillis and Dixon, 1991; Schoch et al., 2012). Over 100,000 fungal ITS sequences generated by Sanger sequencing are stored in the International Nucleotide Sequence Database (INSD) and/or other databases, providing extensive reference material for identifying endophytic fungal species (Nilsson et al., 2009). PCR was used to identify endophytic fungi from diverse tomato plant parts. Using ITS1 and ITS4 primers, *B. bassiana* was validated based on sequence homology (Leckie, 2002). In another study, fungal-specific ITS1-F and ITS4 primers were used in coffee seedlings and a drop in *B. bassiana* colonisation was detected. This may be due to competition with other endophytes in coffee plants, such as *Alternaria* sp. and *Chaetomium sp* (Posada et al., 2007). Potent endophyte isolates of *Aspergillus nidulans* were reported with larvicidal activity against *Spodoptera littoralis* larvae on the sequencing of the flanking ITS regions (El-Sayed et al., 2020). A similar molecular approach was employed to identify 15 native EEPF species isolated from two insect hosts viz., *Hypera postica* and *Gasteracantha fornicata* to study their pathogenicity against the apple blossom beetle (ABB). In addition to molecular characterization, ITS region based phylogenetic analysis of the obtained isolates was also performed using MEGA (Kumar et al., 2008) for the identification of the isolates. The results showed that all the isolates of *B. bassiana* have significant effective activity against ABB adults (Uçar et al., 2022). The ITS (ITS4 and ITS5) sequence analysis of the entomopathogenic fungal isolates from cocoa seedling tissues were carried out and three naturally occurring fungal endophytes viz., *Fusarium redolens*, *Trichoderma asperellum, F. solani, B. bassiana, Metarhizium* sp. and *Hypocrea* sp were identified using molecular techniques. The latter three were reported to show high virulence against termites (*Odontotermes* sp.) (Ambele et al., 2020). 18s rDNA region of ribosomal RNA is another molecular marker broadly applied in molecular fingerprinting studies of fungi. *B. bassiana* isolated from *Atractylodes lancea* was identified based on the conserved sequences in the 18S rDNA (Lv et al., 2014).

### 6.2 Transcriptomics approaches

Transcriptome analysis that is useful for identifying gene function (Iyer et al., 1999) (Yuan et al., 2019) include microarrays that represent almost exclusively mRNAs, i.e. genes translated into proteins. Microarray approach has now been replaced by RNA-Seq, a high-throughput RNA sequencing method (Wang et al., 2009). Transcriptome study of EEPF can help discover its secondary metabolites in two ways. Firstly, these methods can highlight co-regulated gene clusters which comprise such clusters that are separately involved in the synthesis of various secondary metabolites, but whose expression is linked. Secondly, transcriptome techniques can be applied to understand the relationships between genes and secondary metabolites or between genes and active traits (Mao et al., 2015; Cheng et al., 2020). Transcriptome analyses have also revealed the shifting of endophytes lifestyle from biotrophic to necrotrophic during adverse conditions such as reduced carbohydrate levels in bud (Ribeiro et al., 2020). The effects of two *B. bassiana* strains (BG11, FRh2) on *Arabidopsis thaliana* growth and resistance to two herbivore species (aphid, *Myzus persicae* and diamond back moth, *Plutella xylostella*) were studied (Raad et al., 2019) using transcriptomic and metabolomics approaches through microarrays, and upregulation and downregulation of defense-related phytohormones and glucosinolates (GLSs). Root injection with *B. bassiana* BG11 increased plant growth, while FRh2 did not. Both *Beauveria* strains showed no significant effect on *Myzus persicae* population increase or *Plutella xylostella* growth. Metabolomics microarray investigations of leaves from endophyte-inoculated *A. thaliana* showed transcriptional reprogramming of plant defence pathways, with strain-specific changes in the expression of genes linked to pathogenesis, phytoalexin, jasmonic (JA), and salicylic acid (SA) signalling pathways. *B. bassiana* colonisation did not increase JA, SA, or leaf GLSs profiles, which Brassicas use for defence. Thus, EEPF-plant associations can increase biomass and sesquiterpenoids accumulation in *A. lancea* by increasing photosynthesis efficiency, sink expansion (glycolysis and tricarboxylic acid cycle), and metabolic flux (sesquiterpenoids biosynthesis pathway). This study will help clarify plant-EEPF interactions (Yuan et al., 2018).

### 6.3 Proteomics approaches

Besides the aforementioned technologies, the fungal secondary metabolome can also be studied from a proteomics perspective. Recent proteomic analyzes have paved the way for identifying important biomarkers and are able to explain post-transcriptional modifications that can occur during the synthesis of secondary fungal metabolites. Proteomics can also be used to study the fungal secondary metabolome. Recent proteomic analyses have helped discover significant biomarkers and explain post-transcriptional alterations during secondary fungal metabolite production. NRPS, PKS, DMATS, and 2D polyacrylamide gel electrophoresis (PAGE)-LC-MS/MS can be utilised to understand the secondary metabolome and protein alterations. Proteomics can be an effective technique for understanding plant-microbe interactions, despite the complexity of biological materials. Gómez-Vidal et al. (2009) studied the proteomic response of date palm to the endophytic colonization by various EEPF which included *B. bassiana*, *L. dimorphum*, and *L. psalliotae* isolated from infected *Thaumetopoea pityocampa, Saissetia oleae* and *P. marlatti*. Peptides were identified from MS/MS data used to search the nr-NCBI database using the MASCOT software (http://www.matrixscience.com). They identified proteins encoding R genes, proteins associated with stress responses, and smHSPs, to be differentially expressed in infected date palm plants as compared to healthy leaves. These proteins could play a role in plant defence against both biotic and abiotic stress. The study suggested that endophytic colonization of date palm tissue by EEPFs induces a plant defense response, possibly by alerting the plant’s innate immune system (Gómez-Vidal et al., 2009). Further molecular analysis studies conducted on plants infected with EEPFs revealed that endophytes induce important changes in plant metabolism, even if the plants do not show symptoms of endophytic infection. To study low abundance proteins, isobaric tags for relative and absolute quantification (iTRAQ) like strategies have been developed (Wiese et al., 2007; Taylor et al., 2008) which can help decipher the post-transcriptional modifications and proteomics experiments. However, recent advances in metabolomics have provided a facile method to directly detect secondary metabolites and also account for post-transcriptional as well as post-translational modifications. Gómez-Vidal et al. (2009) studied the molecular interactions between three EEPFs viz., *B. bassiana, Lecanicillium dimorphum*, *Lecanicillium psalliotae* and the date palm (*Phoenix dactylifera*) using proteomics techniques.

### 6.4 Metabolomics approaches

Mass spectrometry is used in metabolomics to discover secondary metabolites in microbial cultures and de-duplicate molecules (MS). No separation or purification of fungal cultures is required before analysis therefore, enormous data sets are accessible. To organise MS/MS data by spectrum similarity, comparable sequences are grouped and representative sequences are identified. MS-based molecular cross-linking de-duplicates complex chemical samples like natural products. GNPS (Global Natural Products Social Molecular Networking) archives and makes available processed MS/MS spectrum data (Wang et al., 2016). Together, molecular bridging and GNPS can aid in the identification of certain chemical classes and substances, allowing researchers to better prioritise samples for follow-up analysis. The effects of *B. bassiana* inoculation on lettuce plant development, tissue nutrient content, and proximate composition were investigated, and it was observed that the *B. bassiana* strain induced a mean insect death of 78% at the maximum dose (1 × 10^8^ conidia mL^−1^). Up to 76% of plants were endophytically colonised by *B. bassiana* when exposed to 1 x 108 conidia mL^-1^. Endophytic colonisation had a substantial effect on the plant’s tissue carbon content, which was also connected with the lettuce plants’ antioxidant capacity. Plants treated with *B. bassiana* had higher FRAP (Ferric ion reducing antioxidant power) and TEAC (Trolox equivalent antioxidant capacity) (antioxidants) than those not treated. Further, phytochemical and proximate investigations may shed light on the plant tissue’s macronutrient, micronutrient, and antioxidant composition. The connection between carbon concentration and antioxidant ability may be further understood through metabolomics research in the future (Macuphe et al., 2021).

## 7 Conclusion and future perspectives

EEPFs protect plants from insects, parasitic nematodes and disease pathogens; they promote nutrient uptake, and improve abiotic stress tolerance. Understanding how EEPFs promote nitrogen uptake in host plants might help organic and inorganic fertiliser users save money. EEPFs’ ability to improve plant tolerance to abiotic stresses like heat, salt, and drought can add a new dimension to their interaction with host plants and could be explored or used in agriculture not only to mitigate pests and/or diseases under climate change conditions, but also as an alternative to EPF auto-distribution in inundating applications. Because EPFs applied as inundative sprays for short-term pest control are susceptible to environmental factors, EEPFs residing within plant tissues can help overcome this limitation. When successfully established as endophytes in plants, EPFs can provide long-term, sustainable protection against pests and diseases in horticultural crops. Multi-omics data, including downstream signalling processes, can reveal their nutri-metabolic role as a sustainable pest control tool. The review focuses on the EEPF-plant beneficial relationship on tritrophic interaction, mode of colonisation in diverse horticultural crops, and molecular and biochemical interactions involved in EEPF-mediated insect and natural enemy performance. The current review suggests the following conclusions and future developments.

### 7.1 Qualitative and quantitative alteration of host plant nutrients

It has been reported that EEPF infested plants impact herbivory and the feeding and survival of natural enemies. Through altered VOCs produced by EEPF- colonised plants, EEPF- plant colonisation considerably affects and modulates the insects’ preference, performance, as well as natural enemies’ functional and numerical host finding efficiency. Endophytic fungus-inoculated plants have a distinct profile of volatile organic chemicals than endophyte-free plants, which attracts more insect herbivores. The ecological effect of EEPF in the host plant-insect-natural enemy interaction must be described.

### 7.2 Enhancing nutritional quality of plants

Certain growth and development promoting factors that are caused by EEPF colonisation include nutritional enrichment of plants, morpho-physiological development and tolerance and/or sustenance of varied biotic and/or abiotic challenges and increasing leaf cholorophyll contents.

### 7.3 Increasing constitutive and induced plant defense

Although plants have morpho-chemical constitutive and induced defence mechanisms against various sucking and chewing pests, EEPF association has been shown to increase the protective layers of the plants, promoting both intrinsic and extrinsic resistance.

### 7.4 Constraints in wider application

Though EEPF colonisation has been recorded intracellularly, intercellularly, locally, systemically, and even vertically, research on the artificial establishment of EPF in plants for endophytic colonisation is still in its infancy for pest management. Furthermore, when compared to inundative biological control, EEPFs require a lower inoculum dose, which can be beneficial to farmers in the long run. Pest management in today’s world requires plant protection options that are both economical and environmentally sustainable; EPF can certainly assist. The inclusion of EPF in plants as endophytes seems like a highly fascinating and promising strategy in horticulture crops, but its wider use has been hampered by certain climatically unfavourable situations. Commercially, supplying pre-colonized tomato plants through seedling inoculation may be a good alternative (Silva et al., 2020). EEPF inoculation in horticultural crops may be preferable to chemical applications because of its potential for long-term economic and ecological benefits. When comparing the cost-benefit ratio of different biocontrol methods, such as introduction (classical biological control), augmentation, and conservation, the cost-benefit ratio for classical biological control is extremely favourable (1:250), while the cost-benefit ratio for augmentative control is comparable to that of insecticides (1:2-1:5), with much lower development costs (Bale et al., 2008). EEPFs that establish in the host plants frequently yield larger economic returns than a pesticide application, although the initial stages of identification, isolation and characterization and inoculation methods might be more expensive than for pesticides. In contrast to what has been reported in the past, the danger of resistance and undesirable side effects is higher in chemical control, while the profit per unit of money invested and specificity are higher for biocontrol agents (van Lenteren, 1997; Bale et al., 2008). Although this has not yet been investigated, it is possible to compare the pros and downsides of using pesticides against inoculation EEPFs on horticultural crops.

### 7.5 Lesser explored areas

Despite extensive research on EEPFs, only a few species have been investigated. Because of the scarcity of research in this field, it is often assumed that there are thousands more unknown EEPFs. As a result, there is an urgent need to identify and assess these unknown EEPF strains. Molecular methods and bioinformatics tools may be useful in uncovering this hidden treasure. Studies in the future may concentrate on isolating, identifying, and characterising EEPFs, as well as providing qualitative and quantitative estimates, elucidating structures, and screening the structure-activity link of biologically active molecules.

### 7.6 EEPF study beyond omics

Many bioactive agrochemicals are derived from secondary metabolites produced by endophytes. The current reliance on plants as a source of bioactive compounds can be reduced by the discovery of drugs based on natural products through the EEPF. Fungal endophytes may represent a substantial untapped resource of bioactive natural products/biotic insecticides, as their genomes can be sequenced to reveal previously unknown specialised metabolites and their production methods. In most cases, evidence of endophyte-host mutualism studies were only confined to *Beauveria sp* and *Metarrhizium* sp. There are still many uncovered areas and undiscovered genera of EEPFs, that could potentially serve to understand the intricate yet complicate relationships among host plants- EEPF colonisation on insect-natural enemy interaction. Further studies on the involvement of different receptors, patterns as well as effector molecules that lead to diverse physiological mechanisms in endophytes colonisation, intra and interplant movement of fungal inoculum, EEPF- plant association on insect abundance, survival, preference and response of natural enemies are limited. Additionally, an in-depth knowledge of the abiotic and biotic variables that influence the insecticidal action of EPF and their endophytic behaviour is required for the actual introduction of EEPFs into the IPM programmes.

Though EEPFs have been the subject of a great deal of research, only a small fraction of species have been examined thus far. Due to a lack of investigation, it is widely assumed that there are still thousands of endophytes that have yet to be discovered. Consequently, identifying and assessing these unidentified endophytic strains of EEPFs is of utmost importance.

## Author contributions

IS, TB, PM, SnM, AP, SuM, AP, PA: Conceptualization, writing, original draft preparation, preparation of tables and supervision; A, VS, DM, JK, NM, PB, DK, TaB: Preparation of figures, supervision, review and editing. All authors contributed to the article and approved the submitted version.

## References

[B1] Akello J. Dubois T. Coyne D. Kyamanywa S. (2008). Effect of endophytic *Beauveria bassiana* on populations of the banana weevil, *Cosmopolites sordidus*, and their damage in tissue–cultured banana plants. Entomol Exp. Appl. 129 (2), 157–165. doi: 10.1111/j.1570-7458.2008.00759.x

[B2] Akello J. Sikora R. (2012). Systemic acropedal influence of endophyte seed treatment on *Acyrthosiphon pisum* and *Aphis fabae* offspring development and reproductive fitness. Biol. Control. 61 (3), 215–221. doi: 10.1016/j.biocontrol.2012.02.007

[B3] Akutse K. S. Fiaboe K. K. M. Van Den Berg J. Ekesi S. Maniania N. K. (2014). Effects of endophyte colonization of *Vicia faba* (Fabaceae) plants on the life-history of leafminer parasitoids *Phaedrotoma scabriventris* (Hymenoptera: Braconidae) and *Diglyphus isaea* (Hymenoptera: Eulophidae). PloS One 9 (10), e109965. doi: 10.1371/journal.pone.0109965 25338084 PMC4206285

[B4] Akutse K. S. Maniania N. K. Fiaboe K. K. M. Van den Berg J. Ekesi S. (2013). Endophytic colonization of *Vicia faba* and *Phaseolus vulgaris* (Fabaceae) by fungal pathogens and their effects on the life-history parameters of *Liriomyza huidobrensis* (Diptera: Agromyzidae). Fungal Ecol. 6 (4), 293–301. doi: 10.1016/j.funeco.2013.01.003

[B5] Aldaghi M. Allahyari H. Hosseininaveh V. Behboudi K. (2021). Effect of *Trichoderma harzianum* Tr6 in inducing resistance in tomato against *Trialeurodes vaporariorum* (Hem: Aleyodidae). Plant Prot (Scientific J. Agriculture). 44 (3), 107–117. doi: 2021-10.22055/PPR.2021.17128

[B6] Allegrucci N. Velazquez M. S. Russo M. L. Pérez E. Scorsetti A. C. (2017). Endophytic colonisation of tomato by the entomopathogenic fungus *Beauveria bassiana*: The use of different inoculation techniques and their effects on the tomato leafminer *Tuta absoluta* (Lepidoptera: Gelechiidae). J. Plant Prot. Res. 57 (4), 205–211. doi: 10.1515/jppr-2017-0045

[B7] Altschul S. F. Gish W. Miller W. Myers E. W. Lipman D. J. (1990). Basic local alignment search tool. J. Mol. Biol. 215 (3), 403–410. doi: 10.1016/S0022-2836(05)80360-2 2231712

[B8] Ambele C. F. Ekesi S. Bisseleua H. D. B. Babalola O. O. Khamis F. M. Djuideu C. T. L.. (2020). Entomopathogenic fungi as endophytes for biological control of subterranean termite pests attacking cocoa seedlings. J. Fungi 6 (3), 126. doi: 10.3390/jof6030126 PMC7558646 32764446

[B9] Andolfi A. Maddau L. Basso S. Linaldeddu B. T. Cimmino A. Scanu B.. (2014). Diplopimarane, a 20-nor-ent-pimarane produced by the oak pathogen diplodia quercivora. J. Nat. Pro. 77 (11), 2352–2360. doi: 10.1021/np500258r 25365236

[B10] Arab Y. A. El-Deeb H. M. (2012). The use of endophyte *Beauveria bassiana* for bio-protection of date palm seedlings against red palm weevil and rhizoctonia root-rot disease. Scientific. J. King Faisal Univ (Basic Appl. Sciences). 13 (2), 1433.

[B11] Arnold A. E. Lewis L. C. (2005). Ecology and evolution of fungal endophytes, and their roles against insects. insect-fungal associations. Ecol. Evol. Oxford Univ. Press, 74–96.

[B12] . Available at: http://www.matrixscience.com.

[B13] Bale J. S. Van Lenteren J. C. Bigler F. (2008). Biological control and sustainable food production. Phil Trans. R Soc. B. 363 (1492), 761–776. doi: 10.1098/rstb.2007.2182 17827110 PMC2610108

[B14] Bamisile B. S. Akutse K. S. Siddiqui J. A. Xu Y. (2021). Model application of entomopathogenic fungi as alternatives to chemical pesticides: Prospects, challenges, and insights for next-generation sustainable agriculture. Front. Plant Sci. 12. doi: 10.3389/fpls.2021.741804 PMC8514871 34659310

[B15] Banjo A. D. Lawal O. A. Fapojuwo O. E. Songonuga E. A. (2003). Farmers’ knowledge and perception of horticultural insect pest problems in southeastern Nigeria. Afr J. Biotechnol. 2 (11), 434–437. doi: 10.5897/AJB2003.000-1087

[B16] Barelli L. Moonjely S. Behie S. W. Bidochka M. J. (2016). Fungi with multifunctional lifestyles: Endophytic insect pathogenic fungi. Plant Mol. Biol. 90 (6), 657–664. doi: 10.1007/s11103-015-0413-z 26644135

[B17] Baudoin E. Benizri E. Guckert A. (2003). Impact of artificial root exudates on the bacterial community structure in bulk soil and maize rhizosphere. Soil Biol. Biochem. 35 (9), 1183–1192. doi: 10.1016/S0038-0717(03)00179-2

[B18] Behie S. W. Bidochka M. J. (2014). Ubiquity of insect-derived nitrogen transfer to plants by endophytic insect- pathogenic fungi: An additional branch of the soil nitrogen cycle. Appl. Environ. Microbiol. 80 (5), 1553–1560. doi: 10.1128/AEM.03338-13 24334669 PMC3957595

[B19] Behie S. W. Zelisko P. M. Bidochka M. J. (2012). Endophytic insect-parasitic fungi translocate nitrogen directly from insects to plants. Science 336, 1576–1577. doi: 10.1126/science.1222289 22723421

[B20] Behie S. W. Jones S. J. Bidochka M. J. (2014). Plant tissue localization of the endophytic insect pathogenic fungi *Metarhizium* and Beauveria. Fungal Ecol. 13, 112–119. doi: 10.1016/j.funeco

[B21] Behie S. W. Jones S. J. Bidochka M. J. (2015). Plant tissue localization of the endophytic insect pathogenic fungi *Metarhizium* and *Beauveria* . Fungal Ecol. 13, 112–114. doi: 10.1016/j.funeco.2014.08.001

[B22] Behie S. W. Moreira C. C. Sementchoukova I. Barelli L. Zelisko P. M. Bidochka M. J. (2017). Carbon translocation from a plant to an insect-pathogenic endophytic fungus. Nat. Commun. 8 (1), 14245. doi: 10.1038/ncomms14245 28098142 PMC5253661

[B23] Bing L. A. Lewis L. C. (1991). Suppression of *Ostrinia nubilalis* (Hübner) (Lepidoptera: Pyralidae) by endophytic *Beauveria bassiana* (Balsamo) vuillemin. Environ. Entomol. 20 (4), 1207–1211. doi: 10.1093/ee/20.4.1207

[B24] Bokhorst S. Bjerke J. W. Davey M. P. Taulavuori K. Laine K. Callaghan K. V.. (2010). Impacts of extreme winter warming events on plant physiology in a sub-Arctic heath community. Physiol. Plant 140, 128.e140. doi: 10.1111/j.1399-3054.2010.01386.x 20497369

[B25] Boukal D. S. Bideault A. Carreira B. M. Sentis A. (2019). Species interactions under climate change: Connecting kinetic effects of temperature on individuals to community dynamics. Curr. Opin. Insect Sci. 35, 88–95. doi: 10.1016/j.cois.2019.06.014 31445412

[B26] Cao L. X. You J. L. Zhou S. N. (2002). Endophytic fungi from musa acuminata leaves and roots in south China. World J. Microbiol. Biotechnol. 18 (2), 169–171. doi: 10.1023/A:1014491528811

[B27] Cavicchioli R. Ripple W. J. Timmis K. N. Azam F. Bakken L. R. Baylis M.. (2019). Scientists’ warning to humanity: Microorganisms and climate change. Nat. Rev. Microbiol. 17 (9), 569–586. doi: 10.1038/s41579-019-0222-5 31213707 PMC7136171

[B28] Chavali A. K. Rhee S. Y. (2018). Bioinformatics tools for the identification of gene clusters that biosynthesize specialized metabolites. Brief Bioinform. 19 (5), 1022–1034. doi: 10.1093/bib/bbx020 28398567 PMC6171489

[B29] Cheng J. T. Cao F. Chen X. A. Li Y. Q. Mao X. M. (2020). Genomic and transcriptomic survey of an endophytic fungus *Calcarisporium arbuscula* NRRL 3705 and potential overview of its secondary metabolites. BMC Genomics 21 (1), 424. doi: 10.1186/s12864-020-06813-6 32580753 PMC7315530

[B30] Chen X. Li L. Hu Q. Zhang B. Wu W. Jin F.. (2015). Expression of dsRNA in recombinant isaria fumosorosea strain targets the TLR7 gene in *Bemisia tabaci* . BMC Biotechnol. 15, 64. doi: 10.1186/s12896-015-0170-8 26198409 PMC4509747

[B31] Chidawanyika F. Mudavanhu P. Nyamukondiwa C. (2019). Global climate change as a driver of bottom-up and top-down factors in agricultural landscapes and the fate of host-parasitoid interactions. Front. Ecol. Evol. 7. doi: 10.3389/fevo.2019.00080

[B32] Cimermancic P. Medema M. H. Claesen J. Kurita K. Wieland Brown L. C. W. Mavrommatis K.. (2014). Insights into secondary metabolism from a global analysis of prokaryotic biosynthetic gene clusters. Cell. 158 (2), 412–421. doi: 10.1016/j.cell.2014.06.034 25036635 PMC4123684

[B33] Clancy K. M. Price P. W. (1987). Rapid herbivore growth enhances enemy attack: Sublethal plant defenses remain a paradox. Ecolology. 68 (3), 733–737. doi: 10.2307/1938479

[B34] Clark C. L. Miller J. D. Whitney N. J. (1989). Toxicity of conifer needle endophytes to spruce budworm. Mycol Res. 93 (4), 508–512. doi: 10.1016/S0953-7562(89)80044-9

[B35] Cord-Landwehr S. Melcher R. L. Kolkenbrock S. Moerschbacher B. M. (2016). A chitin deacetylase from the endophytic fungus *Pestalotiopsis* sp. efficiently inactivates the elicitor activity of chitin oligomers in rice cells. Sci. Rep. 6 (1), 38018. doi: 10.1038/srep38018 27901067 PMC5128826

[B36] Cory J. S. Ericsson J. D. (2010). Fungal entomopathogens in a tritrophic context. BioControl. 55 (1), 75–88. doi: 10.1007/s10526-009-9247-4

[B37] Cosme M. Lu J. Erb M. Stout M. J. Franken P. Wurst S. (2016). A fungal endophyte helps plants to tolerate root herbivory through changes in gibberellin and jasmonate signaling. New Phytol. 211 (3), 1065–1076. doi: 10.1111/nph.13957 27061745 PMC5071772

[B38] Crandall S. G. Gold K. M. Jiménez-Gasco M. D. M. Filgueiras C. C. Willett D. S. A. (2020). Multi-omics approach to solving problems in plant disease ecology. PloS One 15 (9), e0237975. doi: 10.1371/journal.pone.0237975 32960892 PMC7508392

[B39] Dash C. K. Bamisile B. S. Keppanan R. Qasim M. Lin Y. Islam S. U.. (2018). Endophytic entomopathogenic fungi enhance the growth of *Phaseolus vulgaris* l. (Fabaceae) and negatively affect the development and reproduction of *Tetranychus urticae* Koch (Acari: Tetranychidae). Microb. Pathog. 125, 385–392. doi: 10.1016/j.micpath.2018.09.044 30290267

[B40] Dinsdale E. A. Edwards R. A. Hall D. Angly F. Breitbart M. Brulc J. M.. (2008). Functional metagenomic profiling of nine biomes. Nature 452 (7187), 629–632. doi: 10.1038/nature06810 18337718

[B41] Di Lelio L. Salvatore M. M. Della Greca M. Mahamedi A. E. Alves A. Berraf-Tebbal A.. (2022). Defensive mutualism of endophytic fungi: Effects of sphaeropsidin a against a model lepidopteran pest. Chem. Proc. 10 (1), 42. doi: 10.3390/IOCAG2022-12216

[B42] Elena G. J. Beatriz P. J. Alejandro P. Lecuona R. E. (2011). *Metarhizium anisopliae* (Metschnikoff) sorokin promotes growth and has endophytic activity in tomato plants. Adv. Biol. Res. 5 (1), 22–27.

[B43] El-Sayed A. S. A. Moustafa A. H. Hussein H. A. El-Sheikh A. A. El-Shafey S. N. Fathy N. A. M.. (2020). Potential insecticidal activity of *Sarocladium strictum*, an endophyte of *Cynanchum acutum*, against *Spodoptera littoralis*, a polyphagous insect pest. Biocatal Agric. Biotechnol. 24, 101524. doi: 10.1016/j.bcab.2020.101524

[B44] Etesami H. Jeong B. R. (2018). Silicon (Si): Review and future prospects on the action mechanisms in alleviating biotic and abiotic stresses in plants. Ecotoxicol Environ. Saf. 147, 881–896. doi: 10.1016/j.ecoenv.2017.09.063 28968941

[B45] Evans H. C. Holmes K. A. Thomas S. E. (2003). Endophytes and mycoparasites associated with an indigenous forest tree, *Theobroma gileri*, in Ecuador and a preliminary assessment of their potential as biocontrol agents of cocoa diseases. Mycol Prog. 2 (2), 149–160. doi: 10.1007/s11557-006-0053-4

[B46] Fan Y. Liu X. Keyhani N. O. Tang G. Pei Y. Zhang W.. (2017). Regulatory cascade and biological activity of *Beauveria bassiana* oosporein that limits bacterial growth after host death. Proc. Natl. Acad. Sci. U S A. 114 (9), E1578–E1586. doi: 10.1073/pnas.1616543114 28193896 PMC5338512

[B47] Fanning P. D. Grieshop M. J. Isaacs R. (2018). Efficacy of biopesticides on spotted wing drosophila, *Drosophila suzukii* matsumura in fall red raspberries. J. Appl. Entomol. 142 (1–2), 26–32. doi: 10.1111/jen.12462

[B48] Flynn D. F. B. Gogol-Prokurat M. Nogeire T. Molinari N. Richers B. T. Lin B. B.. (2009). Loss of functional diversity under land-use intensification across multiple taxa. Ecol. Lett. 12 (1), 22–33. doi: 10.1111/j.1461-709 19087109

[B49] Food and Agriculture Organization (2008). Climate change and food security: A framework document; food and agricultural 689 organization of united nations (Rome, Italy).

[B50] Gao Q. Jin K. Ying S. H. Zhang Y. Xiao G. Shang Y.. (2011). Genome sequencing and comparative transcriptomics of the model entomopathogenic fungi *Metarhizium anisopliae* and *M. acridum* . PloS Genet. 7 (1), e1001264. doi: 10.1371/journal.pgen.1001264 21253567 PMC3017113

[B51] Garrido-Jurado I. Resquín-Romero G. Amarilla S. P. Ríos-Moreno A. Carrasco L. Quesada-Moraga E. (2017). Transient endophytic colonization of melon plants by entomopathogenic fungi after foliar application for the control of *Bemisia tabaci* gennadius (Hemiptera: Aleyrodidae). J. Pest Sci. 90 (1), 319–330. doi: 10.1007/s10340-016-0767-2

[B52] Gautam S. MohanKumar S. Kennedy J. S. (2016). Induced host plant resistance in cauliflower by *Beauveria bassiana* . J. Entomol Zool Stud. 4 (2), 476–482.

[B53] GME (2022). Available at: https://www.globalmarketestimates.com/market-report/horticulture-market-3414.

[B54] Gómez-Vidal S. Salinas J. Tena M. Lopez-Llorca L. V. (2009). Proteomic analysis of date palm (*Phoenix dactylifera* l.) responses to endophytic colonization by entomopathogenic fungi. Electrophoresis. 30 (17), 2996–3005. doi: 10.1002/elps.200900192 19676091

[B55] González-Mas N. Cuenca-Medina M. Gutiérrez-Sánchez F. Quesada-Moraga E. (2019a). Bottom-up effects of endophytic *B. bassiana* on multitrophic interactions between the cotton aphid, *Aphis gossypii*, and its natural enemies in melon. J. Pest Sci. 92 (3), 1271–1281. doi: 10.1007/s10340-019-01098-5

[B56] González-Mas N. Sánchez-Ortiz A. Valverde-García P. Quesada-Moraga E. (2019b). Effects of endophytic entomopathogenic ascomycetes on the life-history traits of *Aphis gossypii* glover and its interactions with melon plants. Insects 10 (6), 165. doi: 10.3390/insects10060165 31185669 PMC6627330

[B57] Greenfield M. Gómez-Jiménez M. I. Ortiz V. Vega F. E. Kramer M. Parsa S. (2016). *Beauveria bassiana* and *Metarhizium anisopliae* endophytically colonize cassava roots following soil drench inoculation. Biol. Control. 95, 40–48. doi: 10.1016/j.biocontrol.2016.01.002 27103778 PMC4825668

[B58] Gunatilaka A. A. (2006). Natural products from plant-associated microorganisms: Distribution, structural diversity, bioactivity, and implications of their occurrence. J. Nat. Prod. 69 (3), 509–526. doi: 10.1021/np058128n 16562864 PMC3362121

[B59] Gurulingappa P. McGee P. A. Sword G. (2011). Endophytic *Lecanicillium lecanii* and *Beauveria bassiana* reduce the survival and fecundity of *Aphis gossypii* following contact with conidia and secondary metabolites. Crop Prot. 30 (3), 349–353. doi: 10.1016/j.cropro.2010.11.017

[B60] Gurulingappa P. Sword G. A. Murdoch G. McGee P. A. (2010). Colonization of crop plants by fungal entomopathogens and their effects on two insect pests when in planta. Biolo control. 55 (1), 34–41. doi: 10.1016/j.biocontrol.2010.06.011

[B61] Hanan A. Basit A. Nazir T. Majeed M. Z. Qiu D. (2020). Anti-insect activity of a partially purified protein derived from the entomopathogenic fungus *Lecanicillium lecanii* (Zimmermann) and its putative role in a tomato defense mechanism against green peach aphid. J. Invertebr Pathol. 170, 107282. doi: 10.1016/j.jip.2019.107282 31759949

[B62] Harith-Fadzilah N. Abd Ghani I. Hassan M. (2021). Omics-based approach in characterising mechanisms of entomopathogenic fungi pathogenicity: A case example of *Beauveria bassiana* . J. King Saud Univ Sci. 33 (2), 101332. doi: 10.1016/j.jksus.2020.101332

[B63] Hartley S. E. Gange A. C. (2009). Impacts of plant symbiotic fungi on insect herbivores: Mutualism in a multitrophic context. Annu. Rev. Entomol. 54, 323–342. doi: 10.1146/annurev.ento.54.110807.090614 19067635

[B64] Hatcher P. E. (1995). Three-way interactions between plant pathogenic fungi, herbivorous insects and their host plants. Biol. Rev. 70 (4), 639–694. doi: 10.1111/j.1469-185X.1995.tb01655.x

[B65] Hillis D. M. Dixon M. T. (1991). Ribosomal DNA: Molecular evolution and phylogenetic inference. Q Rev. Biol. 66 (4), 411–453. doi: 10.1086/417338 1784710

[B66] Hu S. Bidochka M. J. (2021). Root colonization by endophytic insectpathogenic fungi. J. Appl. Microbiol. 130 (2), 570–581. doi: 10.1111/jam.14503 31667953

[B67] Inglis G. D. Goettel M. S. Butt T. M. Strasser H. (2001). “Use of hyphomycetous fungi for managing insect pests,” in Fungi as biocontrol agents. Eds. Butt T. M. Jackson C. Magan N. (CABI Publishing International), 23–69.

[B68] Iyer S. N. Gurujeyalakshmi G. Giri S. N (1999). Effects of pirfenidone on transforming growth factor-β gene expression at the transcriptional level in bleomycin hamster model of lung fibrosis. J. Pharmacol. Exp. Ther. 291, 367–373. doi: 10.1016/j.biocontrol.2017.04.005 10490926

[B69] Jaber L. R. Araj S. E. (2018). Interactions among endophytic fungal entomopathogens (Ascomycota: Hypocreales), the green peach aphid *Myzus persicae* sulzer (Homoptera: Aphididae), and the aphid endoparasitoid *Aphidius colemani* viereck (Hymenoptera: Braconidae). Biol. Control. 116, 53–61. doi: 10.1016/j.biocontrol.2017.04.005

[B70] Jaber L. R. Araj S. E. Qasem J. R. (2018). Compatibility of endophytic fungal entomopathogens with plant extracts for the management of sweetpotato whitefly *Bemesia tabaci* gennadius (Homoptera: Aleyrodidae). Biol. Control. 117, 164–171. doi: 10.1016/j.biocontrol.2017.11.009

[B71] Jaber L. R. Enkerli J. (2017). Fungal entomopathogens as endophytes: Can they promote plant growth? Biocontrol Sci. Technol. 27 (1), 28–41. doi: 10.1080/09583157.2016.1243227

[B72] Jaihan P. Sangdee K. Sangdee A. (2016). Selection of entomopathogenic fungus for biological control of chili anthracnose disease caused by *Colletotrichum* spp. Eur. J. Plant Pathol. 146 (3), 551–564. doi: 10.1007/s10658-016-0941-7

[B73] Jallow M. F. A. Dugassa-Gobena D. Vidal S. (2008). Influence of an endophytic fungus on host plant selection by a polyphagous moth *via* volatile spectrum changes. Arthropod Plant Interact. 2 (1), 53–62. doi: 10.1007/s11829-008-9033-8

[B74] Jensen R. E. Enkegaard A. Steenberg T. (2019). Increased fecundity of aphis fabae on *Vicia faba* plants following seed or leaf inoculation with the entomopathogenic fungus *Beauveria bassiana* . PloS One 14 (10), e0223616. doi: 10.1371/journal.pone.0223616 31589639 PMC6779261

[B75] Jones K. D. (1994). Aspects of the biology and biological control of the European corn borer in north Carolina [PhD thesis] (North Carolina State University).

[B76] Kandalepas D. Blum M. J. Van Bael S. A. (2015). Shifts in symbiotic endophyte communities of a foundational salt marsh grass following oil exposure from the deepwater horizon oil spill. PloS One 10 (4), e0122378. doi: 10.1371/journal.pone.0122378 25923203 PMC4414556

[B77] Khachatourians G. G. Qazi S. S. (2008). “Entomopathogenic fungi: Biochemistry and molecular biology,” in Human and animal relationships. Eds. Brakhage A. A. Zipfel P. F. (Berlin, Heidelberg: Springer), 33–61.

[B78] Khaldi N. Seifuddin F. T. Turner G. Haft D. Nierman W. C. Wolfe K. H.. (2010). Smurf: Genomic mapping of fungal secondary metabolite clusters. Fungal Genet. Biol. 47 (9), 736–741. doi: 10.1016/j.fgb.2010.06.003 20554054 PMC2916752

[B79] Khan A. L. Hamayun M. Khan S. A. Kang S. M. Shinwari Z. K. Kamran M.. (2012). Pure culture of *Metarhizium anisopliae* LHL07 reprograms soybean to higher growth and mitigates salt stress. World J. Microbiol. Biotechnol. 28 (4), 1483–1494. doi: 10.1007/s11274-011-0950-9 22805930

[B80] Kim J. J. Goettel M. S. Gillespie D. R. (2010). Evaluation of *Lecanicillium longisporum*, vertalec® against the cotton aphid, *Aphis gossypii*, and cucumber powdery mildew, *Sphaerotheca fuliginea* in a greenhouse environment. Crop Prot. 29 (6), 540–544. doi: 10.1016/j.cropro.2009.12.011

[B81] Klieber J. Reineke A. (2016). The entomopathogen *Beauveria bassiana* has epiphytic and endophytic activity against the tomato leaf miner *Tuta absoluta* . J. Appl. Entomol. 140 (8), 580–589. doi: 10.1111/jen.12287

[B82] Kumar S. Nei M. Dudley J. Tamura K. (2008). MEGA: A biologist-centric software for evolutionary analysis of DNA and protein sequences. Brief Bioinform. 9 (4), 299–306. doi: 10.1093/bib/bbn017 18417537 PMC2562624

[B83] Kunkel B. A. Grewal P. S. Quigley M. F. (2004). A mechanism of acquired resistance against an entomopathogenic nematode by *Agrotis ipsilon* feeding on perennial ryegrass harboring a fungal endophyte. Biol. Control 29 (1), 100–108. doi: 10.1016/S1049-9644(03)00119-1

[B84] Landa B. B. López-Díaz C. Jiménez-Fernández D. Montes-Borrego M. Muñoz-Ledesma F. J. Ortiz-Urquiza A.. (2013). In-planta detection and monitorization of endophytic colonization by a *Beauveria bassiana* strain using a new-developed nested and quantitative PCR-based assay and confocal laser scanning microscopy. J. Invertebr Pathol. 114 (2), 128–138. doi: 10.1016/j.jip.2013.06.007 23851123

[B85] Leckie B. M. (2002). Effects of beauveria bassiana mycelia and metabolites incorporated into synthetic diet and fed to larval helicoverpa zea; and detection of endophytic beauveria bassiana in tomato plants using PCR and ITS primers. [Masters thesis] (University of Tennessee).

[B86] Li H. Guan Y. Dong Y. Zhao L. Rong S. Chen W.. (2018). Isolation and evaluation of endophytic *Bacillus tequilensis* GYLH001 with potential application for biological control of *Magnaporthe oryzae* . PloS One 13 (10), e0203505. doi: 10.1371/journal.pone.0203505 30379821 PMC6209128

[B87] Litwin A. Nowak M. Różalska S. (2020). Entomopathogenic fungi: Unconventional applications. Rev. Environ. Sci. Bio Technol. 19 (1), 23–42. doi: 10.1007/s11157-020-09525-1

[B88] Lozano-Soria A. Picciotti U. Lopez-Moya F. Lopez-Cepero J. Porcelli F. Lopez-Llorca L. V. (2020). Volatile organic compounds from entomopathogenic and nematophagous fungi, repel banana black weevil (*Cosmopolites sordidus*). Insects. 11 (8), 509. doi: 10.3390/insects11080509 32781701 PMC7469225

[B89] Lv L. X. Wang H. W. Liang X. F. Hao S. J. Du W. Zhu H.. (2014). Effects of different chemotypes and seasonal dynamic variation on species diversity of endophytic fungal communities harbored in *Atractylodes lancea* . Acta Ecol. Sin. 34, 7300–7310.

[B90] Macuphe N. Oguntibeju O. O. Nchu F. (2021). Evaluating the endophytic activities of beauveria bassiana on the physiology, growth, and antioxidant activities of extracts of lettuce (*Lactuca sativa* l.). Plants 10 (6), 1178. doi: 10.3390/plants10061178 34207888 PMC8229626

[B91] Manoussopoulos Y. Mantzoukas S. Lagogiannis I. Goudoudaki S. Kambouris M. (2019). Effects of three strawberry entomopathogenic fungi on the prefeeding behavior of the aphid *Myzus persicae* . J. Insect Behav. 32 (2), 99–108. doi: 10.1007/s10905-019-09709-w

[B92] Mantzoukas S. Lagogiannis I. (2019). Endophytic colonization of pepper (*Capsicum annum*) controls aphids (*Myzus persicae* sulzer). Appl. Sci. 9 (11), 2239. doi: 10.3390/app9112239

[B93] Mao X. M. Zhan Z. J. Grayson M. N. Tang M. C. Xu W. Li Y. Q.. (2015). Efficient biosynthesis of fungal polyketides containing the dioxabicyclo-octane ring system. J. Am. Chem. Soc 137 (37), 11904–11907. doi: 10.1021/jacs.5b07816 26340065 PMC4903023

[B94] Martínez-Medina A. Appels F. V. W. van Wees S. C. M. (2017). Impact of salicylic acid- and jasmonic acid-regulated defences on root colonization by *Trichoderma harzianum* T-78. Plant Signal Behav. 12 (8), e1345404. doi: 10.1080/15592324.2017.1345404 28692334 PMC5616143

[B95] Martinuz A. Schouten A. Menjivar R. D. Sikora R. A. (2012). Effectiveness of systemic resistance toward *Aphis gossypii* (Homoptera: Aphididae) as induced by combined applications of the endophytes *Fusarium oxysporum* Fo162 and *Rhizobium etli* G12. Bioll Control. 62 (3), 206–212. doi: 10.1016/j.biocontrol.2012.05.006

[B96] Medema M. H. Blin K. Cimermancic P. De Jager V. Zakrzewski P. Fischbach M. A.. (2011). antiSMASH: Rapid identification, annotation and analysis of secondary metabolite biosynthesis gene clusters in bacterial and fungal genome sequences. Nucleic Acids Res. 39(Web Server issue) (Web Server issue), W339–W346. doi: 10.1093/nar/gkr466 21672958 PMC3125804

[B97] Mendoza-Mendoza A. Zaid R. Lawry R. Hermosa R. Monte E. Horwitz B. A.. (2018). Molecular dialogues between *Trichoderma* and roots: Role of the fungal secretome. Fungal Biol. Rev. 32 (2), 62–85. doi: 10.1016/j.fbr.2017.12.001

[B98] Mercado-Blanco J. Lugtenberg B. (2014). Biotechnological applications of bacterial endophytes. Curr. Biotechnol. 3 (1), 60–75. doi: 10.2174/22115501113026660038

[B99] Miranda-Fuentes P. Quesada-Moraga E. Aldebis H. K. Yousef-Naef M. (2020). Compatibility between the endoparasitoid *Hyposoter didymator* and the entomopathogenic fungus *Metarhizium brunneum*: A laboratory simulation for the simultaneous use to control spodoptera littoralis. Pest Manag Sci. 76 (3), 1060–1070. doi: 10.1002/ps.5616 31515940

[B100] Miranda-Fuentes P. Yousef-Yousef M. Valverde-Garcia P. Rodríguez-Gómez I. M. Garrido-Jurado I. Quesada-Moraga E. (2021). Entomopathogenic fungal endophyte-mediated tritrophic interactions between *Spodoptera littoralis* and its parasitoid *Hyposoter didymator* . J. Pest Sci. 94 (3), 933–945. doi: 10.1007/s10340-020-01306-7

[B101] Moonjely S. Barelli L. Bidochka M. J. (2016). “Insect pathogenic fungi as endophytes,” in Advances in genetics. Ed. St. Leger R. J. (Academic Press), 107–135. doi: 10.1016/bs.adgen.2015.12.004 27131324

[B102] Moraga E. (2021). Entomopathogenic fungal endophyte-mediated tritrophic interactions between Spodoptera littoralis and its parasitoid Hyposoter didymator. PloS one 94 (3), 933–945. doi: 10.1007/s10340-020-01306-7

[B103] Muvea A. M. Meyhöfer R. Subramanian S. Poehling H. M. Ekesi S. Maniania N. K. (2014). Colonization of onions by endophytic fungi and their impacts on the biology of Thrips tabaci. PloS one 9 (9), e108242. doi: 10.1371/journal.pone.0108242 25254657 PMC4177896

[B104] Newman M. A. Sundelin T. Nielsen J. T. Erbs G. (2013). MAMP (microbeassociated molecular pattern) triggered immunity in plants. Front. Plant Sci. 4. doi: 10.3389/fpls.2013.00139 PMC3655273 23720666

[B105] Nilsson R. H. Ryberg M. Abarenkov K. Sjökvist E. Kristiansson E. (2009). The ITS region as a target for characterization of fungal communities using emerging sequencing technologies. FEMS Microbiol. Lett. 296 (1), 97–101. doi: 10.1111/j.1574-6968.2009.01618.x 19459974

[B106] Oerke E. C. Dehne H.-W. (2004). Safeguarding production–losses in major crops and the role of crop protection. Crop Prot 23 (4), 275–285. doi: 10.1016/j.cropro.2003.10.001

[B107] Omacini M. Chaneton E. J. Ghersa C. M. Müller C. B. (2001). Symbiotic fungal endophytes control insect host–parasite interaction webs. Nature 409 (6816), 78–81. doi: 10.1038/35051070 11343116

[B108] Ownley B. H. Pereira R. M. Klingeman W. E. Quigley N. B. Leckie B. M. (2004). “Beauveria bassiana, a dual purpose biocontrol organism, with activity against insect pests and plant pathogens,” in Emerging concepts in plant health management. Eds. Lartey R. Caesar A. (Research Signpost), 256–269.

[B109] Parsa S. Ortiz V. Vega F. E. (2013). Establishing fungal entomopathogens as endophytes: Towards endophytic biological control. J. Vis. Exp. 74, 1–5. doi: 10.3791/50360 PMC3654456 23603853

[B110] Pattemore J. A. Hane J. K. Williams A. H. Wilson B. A. Stodart B. J. Ash G. J. (2014). The genome sequence of the biocontrol fungus *Metarhizium anisopliae* and comparative genomics of *Metarhizium* species. BMC Genomics 15 (1), 660. doi: 10.1186/1471-2164-15-660 25102932 PMC4133081

[B111] Pieterse C. M. J. Dicke M. (2007). Plant interactions with microbes and insects: From molecular mechanisms to ecology. Trends Plant Sci. 12 (12), 564–569. doi: 10.1016/j.tplants.2007.09.004 17997347

[B112] Pimentel M. R. Molina G. Dionísio A. P. Maróstica Junior M. R. Pastore G. M. (2011). The use of endophytes to obtain bioactive compounds and their application in biotransformation process. Biotechnol. Res. Int., 2576286. doi: 10.4061/2011/576286 PMC3042614 21350663

[B113] Poling S. M. Wicklow D. T. Rogers K. D. Gloer J. B. (2008). Acremonium zeae, a protective endophyte of maize produces dihydroresorcylide and 7-hydroxydihydroresorcylides. J. Agric. Food Chem. 56 (9), 3006–3009. doi: 10.1021/jf073274f 18416554

[B114] Posada F. Aime M. C. Peterson S. W. Rehner S. A. Vega F. E. (2007). Inoculation of coffee plants with the fungal entomopathogen *Beauveria bassiana* (Ascomycota: Hypocreales). Mycol Res. 111 (6), 748–757. doi: 10.1016/j.mycres.2007.03.006 17604149

[B115] Powell W. A. Klingeman W. E. Ownley B. H. Gwinn K. D. (2009). Evidence of endophytic beauveria bassiana in seed-treated tomato plants acting as a systemic entomopathogen to larval *Helicoverpa zea* (Lepidoptera: Noctuidae). J. Entomol Sci. 44 (4), 391–396. doi: 10.18474/0749-8004-44.4.391

[B116] Price P. W. Bouton C. E. Gross P. McPheron B. A. Thompson J. N. Weis A. E. (1980). Interactions among three trophic levels: Influence of plants on interactions between insect herbivores and natural enemies. Annu. Rev. Ecol. Syst. 11 (1), 41–65. doi: 10.1146/annurev.es.11.110180.000353

[B117] Puri A. Padda K. P. Chanway C. P. (2016). Evidence of nitrogen fixation and growth promotion in canola (*Brassica napus* l.) by an endophytic diazotroph *Paenibacillus polymyxa* P2b-2R. Biol. Fertil Soils. 52 (1), 119–125. doi: 10.1007/s00374-015-1051-y

[B118] Qayyum M. A. Wakil W. Arif M. J. Sahi S. T. Dunlap C. A. (2015). Infection of *Helicoverpa armigera* by endophytic *Beauveria bassiana* colonizing tomato plants. Biol. Control. 90, 200–207. doi: 10.1016/j.biocontrol.2015.04.005

[B119] Quesada Moraga E. (2020). Entomopathogenic fungi as endophytes: Their broader contribution to IPM and crop production. Biocontrol Sci. Technol. 30 (9), 864–877. doi: 10.1080/09583157.2020.1771279

[B120] Quesada-Moraga E. Landa B. B. Muñoz-Ledesma J. Jiménez-Diáz R. M. Santiago-Alvarez C. (2006). Endophytic colonisation of opium poppy, *Papaver somniferum*, by an entomopathogenic *Beauveria bassiana* strain. Mycopathologia. 161 (5), 323–329. doi: 10.1007/s11046-006-0014-0 16649082

[B121] Quesada-Moraga E. Muñoz-Ledesma F. J. Santiago-Álvarez C. (2009). Systemic protection of the opium poppy, *Papaver somniferum* l., against *Iraella luteipes* (Hymenoptera; cynipidae) by an endophytic strain of *Beauveria bassiana* (Ascomycota; hypocrales). Environ. Entomol. 38 (3), 723–730. doi: 10.1603/022.038.0324 19508781

[B122] Rangel D. E. N. Fernandes E. K. K. Dettenmaier S. J. Roberts D. W. (2010). Thermotolerance of germlings and mycelium of the insect-pathogenic fungus metarhizium spp. and mycelial recovery after heat stress. J. Basic Microbiol. 50 (4), 344–350. doi: 10.1002/jobm.200900430 20586069

[B123] Raya-Díaz S. Sánchez-Rodríguez A. R. Segura-Fernández J. M. Del Campillo M. D. C. Quesada-Moraga E. (2017). Entomopathogenic fungi-based mechanisms for improved fe nutrition in sorghum plants grown on calcareous substrates. PloS One 12 (10), e0185903. doi: 10.1371/journal.pone.0185903 28982140 PMC5628914

[B124] Resquín-Romero G. A. Garrido-Jurado I. Delso C. Ríos-Moreno A. Quesada-Moraga E. (2016). Transient endophytic colonizations of plants improve the outcome of foliar applications of mycoinsecticides against chewing insects. J. Invertebr Pathol. 136, 23–31. doi: 10.1016/j.jip.2016.03.003 26945771

[B125] Ribeiro T. H. C. Fernandes-Brum C. N. de Souza C. R. Dias F. A. N. Almeida-Junior O. D. Regina M. A.. (2020). Transcriptome analyses suggest that changes in fungal endophyte lifestyle could be involved in grapevine bud necrosis. Sci. Rep. 10 (1), 9514. doi: 10.1038/s41598-020-66500-0 32528037 PMC7290027

[B126] Richmond D. S. Kunkel B. A. Somasekhar N. Grewal P. S. (2004). Top-down and bottom-up regulation of herbivores: *Spodoptera frugiperda* turns tables on endophyte-mediated plant defence and virulence of an entomopathogenic nematode. Ecol. Entomol. 29 (3), 353–360. doi: 10.1111/j.1365-2311.2004.00598.x

[B127] Rodriguez R. Durán P. (2020). Natural holobiome engineering by using native extreme microbiome to counteract the climate change effects. Front. bioeng Biotechnol. 8. doi: 10.3389/fbioe.2020.00568 PMC7287022 32582678

[B128] Rodríguez-González A. Casquero P. A. Cardoza R. E. Gutiérrez S. (2019). Effect of trichodiene synthase encoding gene expression in *Trichoderma* strains on their effectiveness in the control of *Acanthoscelides obtectus* . J. Stored Prod Res. 83, 275–280. doi: 10.1016/j.jspr.2019.07.006

[B129] Rondot Y. Reineke A. (2018). Endophytic *Beauveria bassiana* in grapevine vitis vinifera (L.) reduces infestation with piercing-sucking insects. Biol. Control. 116, 82–89. doi: 10.1016/j.biocontrol.2016.10.006

[B130] Saha T. Chandran N. Anu B. C. (2020). Major insect pests of vegetable crops in bihar and their management. J sustain agric (Apple Academic Press), 395–429.

[B131] Sánchez-Rodríguez A. R. Raya-Díaz S. Zamarreño ÁM. García-Mina J. M. del Campillo M. C. Quesada-Moraga E. (2018). An endophytic *Beauveria bassiana* strain increases spike production in bread and durum wheat plants and effectively controls cotton leafworm (*Spodoptera littoralis*) larvae. Biol. Control. 116, 90–102. doi: 10.1016/j.biocontrol.2017.01.012

[B132] Sánchez-Vallet A. Mesters J. R. Thomma B. P. (2015). The battle for chitin recognition in plant-microbe interactions. FEMS Microbiol. Rev. 39 (2), 171–183. doi: 10.1093/femsre/fuu003 25725011

[B133] Schoch C. L. Seifert K. A. Huhndorf S. Robert V. Spouge J. L. Levesque C. A.. (2012). Nuclear ribosomal internal transcribed spacer (ITS) region as a universal DNA barcode marker for fungi. Proc. Natl. Acad. Sci. U S A. 109 (16), 6241–6246. doi: 10.1073/pnas.1117018109 22454494 PMC3341068

[B134] Schulz B. Boyle C. (2005). The endophytic continuum. Mycol Res. 109 (6), 661–686. doi: 10.1017/s095375620500273x 16080390

[B135] Silva A. C. L. Silva G. A. Abib P. H. N. Carolino A. T. Samuels R. I. (2020). Endophytic colonization of tomato plants by the entomopathogenic fungus *Beauveria bassiana* for controlling the south American tomato pinworm, tuta absoluta. CABI. J. Agric. Biol. Sci. 1 (1), 1–9. doi: 10.1186/s43170-020-00002-x

[B136] Shrivastava G. Ownley B. H. Augé R. M. Toler H. Dee M. Vu A. (2015). Colonization by arbuscular mycorrhizal and endophytic fungi enhanced terpene production in tomato plants and their defense against a herbivorous insect. Symbiosis 65 (2), 65–74.

[B137] Starcevic A. Zucko J. Simunkovic J. Long P. F. Cullum J. Hranueli D. (2008). ClustScan: An integrated program package for the semi-automatic annotation of modular biosynthetic gene clusters and in silico prediction of novel chemical structures. Nucleic Acids Res. 36 (21), 6882–6892. doi: 10.1093/nar/gkn685 18978015 PMC2588505

[B138] Sui L. Zhu H. Xu W. Guo Q. Wang L. Zhang Z.. (2020). Elevated air temperature shifts the interactions between plants and endophytic fungal entomopathogens in an agroecosystem. Fungal Ecol. 47, 100940. doi: 10.1016/j.funeco.2020.100940

[B139] Suryanarayanan T. S. (2013). Endophyte research: Going beyond isolation and metabolite documentation. Fungal Ecol. 6 (6), 561–568. doi: 10.1016/j.funeco.2013.09.007

[B140] Suryanarayanan T. S. Shaanker R. U. (2021). Can fungal endophytes fast-track plant adaptations to climate change? Fungal Eco. 50, 101039. doi: 10.1016/j.funeco.2021.101039

[B141] Taylor R. D. Saparno A. Blackwell B. Anoop V. Gleddie S. Tinker N. A.. (2008). Proteomic analyses of *Fusarium graminearum* grown under mycotoxin-inducing conditions. Proteomics. 8 (11), 2256–2265. doi: 10.1002/pmic.200700610 18452225

[B142] Tiwari V. Charuvi D. (2021). “Transgenic horticultural crops for combating abiotic stresses,” in Stress tolerance in horticultural crops (Woodhead Publishing), 301–326.

[B143] Tiwari R. K. Lal M. K. Naga K. C. Kumar R. Chourasia K. N. Kumar D.. (2020). Emerging roles of melatonin in mitigating abiotic and biotic stresses of horticultural crops. Sci. Hortic. 272, 109592. doi: 10.1016/j.scienta.2020.109592

[B144] Tkaczuk C. Harasimiuk M. Król A. Bereś P. K. (2015). The effect of selected pesticides on the growth of entomopathogenic fungi *Hirsutella nodulosa* and *Beauveria bassiana* . J. Ecol. Eng. 16 (3), 177–183. doi: 10.12911/22998993/2952

[B145] Uçar S. Atay T. Yanar Y. (2022). Identification of some native entomopathogenic fungal species and their pathogenicity against apple blossom beetle, *Tropinota* (*Epicometis*) *hirta* (Poda 1761) (Coleoptera: Cetoniidae) adults. Egypt J. Biol. Pest Control. 32 (1), 1–8. doi: 10.1186/s41938-022-00594-8

[B146] Umemura M. Koike H. Nagano N. Ishii T. Kawano J. Yamane N.. (2013). MIDDAS-m: Motif-independent *de novo* detection of secondary metabolite gene clusters through the integration of genome sequencing and transcriptome data. PloS One 8 (12), e84028. doi: 10.1371/journal.pone.0084028 24391870 PMC3877130

[B147] United Nations (2019) World population prospects: Highlights. Available at: http://un.org/development/desa/publications/world-population-prospects-2019-highlights.html.

[B148] van Lenteren J. C. (1997). Benefits and risk of introducing exotic macro-biological control agents into Europe. EPPO Bull. 27 (1), 15–27. doi: 10.1111/j.1365-2338.1997.tb00611.x

[B149] Vega F. E. (2008). Insect pathology and fungal endophytes. J. Invertebr Pathol. 98 (3), 277–279. doi: 10.1016/j.jip.2008.01.008 18406422

[B150] Vega F. E. (2018). The use of fungal entomopathogens as endophytes in biological control: A review. Mycologia 110 (1), 4–30. doi: 10.1080/00275514.2017.1418578 29863999

[B151] Vega F. E. Meyling N. V. Luangsa-ard J. J. Blackwell M. (2012). “Fungal entomopathogens,” in Insect pathology. Eds. Vega F. E. Kaya H. K. (Academic Press), 171–220. doi: 10.1016/B978-0-12-384984-7.00006-3.

[B152] Wang C. S. St. Leger R. J.. (2007). The MAD1 adhesin of Metarhizium anisopliae links adhesion with blastospore production and virulence to insects, and the MAD2 adhesin enables attachment to plants. Eukaryotic Cell 6, 808e816. doi: 10.1128/EC.00409-06 17337634 PMC1899246

[B153] Wang M. Carver J. J. Phelan V. V. Sanchez L. M. Garg N. Peng Y.. (2016). Sharing and community curation of mass spectrometry data with global natural products social molecular networking. Nat. Biotechnol. 34 (8), 828–837. doi: 10.1038/nbt.3597 27504778 PMC5321674

[B154] Wang Z. Gerstein M. Snyder M. (2009). RNA-Seq: A revolutionary tool for transcriptomics. Nat. Rev. Genet. 10 (1), 57–63. doi: 10.1038/nrg2484 19015660 PMC2949280

[B155] Wang X. Zhang X. Liu L. Xiang M. Wang W. Sun X.. (2015). Genomic and transcriptomic analysis of the endophytic fungus *Pestalotiopsis fici* reveals its lifestyle and high potential for synthesis of natural products. BMC Genomics 16 (1), 28. doi: 10.1186/s12864-014-1190-9 25623211 PMC4320822

[B156] Weber T. Rausch C. Lopez P. Hoof I. Gaykova V. Huson D. H.. (2009). CLUSEAN: A computer-based framework for the automated analysis of bacterial secondary metabolite biosynthetic gene clusters. J. Biotechnol. 140 (1–2), 13–17. doi: 10.1016/j.jbiotec.2009.01.007 19297688

[B157] Wei Q. Y. Li Y. Y. Xu C. Wu Y. X. Zhang Y. R. Liu H. (2020). Endophytic colonization by *Beauveria bassiana* increases the resistance of tomatoes against *Bemisia tabaci* . Arthropod Plant Interact. 14 (3), 289–300. doi: 10.1007/s11829-020-09746-9

[B158] Wiese S. Reidegeld K. A. Meyer H. E. Warscheid B. (2007). Protein labeling by iTRAQ: A new tool for quantitative mass spectrometry in proteome research. Proteomics. 7 (3), 340–350. doi: 10.1002/pmic.200600422 17177251

[B159] Xiao G. Ying S. H. Zheng P. Wang Z. L. Zhang S. Xie X. Q.. (2012). Genomic perspectives on the evolution of fungal entomopathogenicity in *Beauveria bassiana* . Sci. Rep. 2 (1), 483. doi: 10.1038/srep00483 22761991 PMC3387728

[B160] Yuan H. G. Wu S. Y. Lei Z. R. Rondon S. I. Gao Y. L.. (2018). Sub-lethal effects of Beauveria bassiana (Balsamo) on field populations of the potato tuber worm Phthorimaea operculella Zeller in China. J. integr. Agric. 17 (4), 911–918. doi: 10.1016/S2095-3119(17)61898-7

[B161] Yuan J. Zhang W. Sun K. Tang M. J. Chen P. X. Li X.. (2019). Comparative transcriptomics and proteomics of *Atractylodes lancea* in response to endophytic fungus gilmaniella sp. AL12 reveals regulation in plant metabolism. Front. Microbiol. 10. doi: 10.3389/fmicb.2019.01208 PMC6546907 31191508

[B162] Zamioudis C. Pieterse C. M. (2012). Modulation of host immunity by beneficial microbes. Mol. Plant Microbe Interact. 25 (2), 139–150. doi: 10.1094/MPMI-06-11-0179 21995763

[B163] Zeidler D. Zähringer U. Gerber I. Dubery I. Hartung T. Bors W.. (2004). Innate immunity in *Arabidopsis thaliana*: Lipopolysaccharides activate nitric oxide synthase (NOS) and induce defense genes. Proc. Natl. Acad. Sci. U S A. 101 (44), 15811–15816. doi: 10.1073/pnas.0404536101 15498873 PMC524824

[B164] Zhang Q. Chen X. Xu C. Zhao H. Zhang X. Zeng G.. (2019). Horizontal gene transfer allowed the emergence of broad host range entomopathogens. Proc. Natl. Acad. Sci. U S A. 116 (16), 7982–7989. doi: 10.1073/pnas.1816430116 30948646 PMC6475382

[B165] Zhang S. Xia Y. X. Kim B. Keyhani N. O. (2011). Two hydrophobins are involved in fungal spore coat rodlet layer assembly and each play distinct roles in surface interactions, development and pathogenesis in the entomopathogenic fungus, *Beauveria bassiana* . Mol. Microbiol. 80 (3), 811–826. doi: 10.1111/j.1365-2958.2011.07613.x 21375591

[B166] Zimmermann G. (2008). The entomopathogenic fungi *Isaria far*inosa (formerly *Paecilomyces farinosus*) and the *Isaria fumosorosea* species complex (formerly paecilomyces fumosoroseus): Biology, ecology and use in biological control. Biocontrol Sci. Technol. 18 (9), 865–901. doi: 10.1080/09583150802471812

